# Single‐track year‐round education for improving academic achievement in U.S. K‐12 schools: Results of a meta‐analysis

**DOI:** 10.1002/cl2.1053

**Published:** 2019-09-24

**Authors:** Dan Fitzpatrick, Jason Burns

**Affiliations:** ^1^ Department of Educational Administration, College of Education Michigan State University East Lansing Michigan

## Abstract

**Background:**

Research shows that over summer break, students forget approximately 1 month of learning in math and reading; furthermore, some studies find that low‐income students lose ground relative to peers. Year‐round education (YRE) redistributes schooldays to shorten summer. Prior analyses pooled single‐track YRE (academic intervention in which all students attend school on a common calendar) and multitrack YRE (fiscal intervention countering overcrowding, in which groups of students attend school on staggered schedules).

**Search Methods:**

Systematic search of 22 online databases in summer 2017 yielded 494 de‐duplicated results; 81 warranted full‐text examination. After applying selection criteria, nine studies met criteria but did not report data that allowed effect size calculation. Thirty studies constituted our analytic sample.

**Selection Criteria:**

Studies needed to be of K‐12 single‐track YRE (not multitrack, not a mix of single‐ and multitrack, and not a study that did not specify track), with no accompanying extended instructional time. Studies needed to be from 2001 to 2016, include outcome data, and include a comparison group.

**Data:**

We extracted 55 math *g*, 58 reading *g*, 29 math odds ratio, and 27 reading odds ratio effect sizes.

**Results:**

Students at single‐track YRE schools show modestly higher achievement in both math and reading—by a magnitude similar to estimates of summer learning loss—but comparable proficiency. Unexpectedly, the effect was no greater for historically disadvantaged students. Math effects may be larger in middle than elementary school, but the reason is unclear. Importantly, studies of schools that shortened summer to the fewest weeks showed the largest effects in both subjects.

## 1 PLAIN LANGUAGE SUMMARY

Single‐track year‐round education modestly improves average math and reading achievement of K‐12 students

### 1.1 The review in brief

Single‐track year‐round education (YRE) is linked with higher average achievement in both math and reading. Achievement gains from single‐track YRE are similar in magnitude to the degree of summer learning loss documented in other studies. However, no difference was found in proficiency rates in either subject. Possible reasons for lack of effect on proficiency are discussed in the review.

### 1.2 What is this review about?

Over the long summer break, students forget some of what they learned during the prior school year. For low‐income students, this “summer learning loss” may be especially large. One policy aimed at decreasing summer learning loss is YRE: redistributing the usual number of school days so that students have more short breaks during the school year, but a much shorter summer vacation. A specific design used to achieve this goal is single‐track YRE, which involves placing all students attending a given school on the same year‐round calendar. This review considers evidence on the effect of single‐track YRE on academic achievement—test scores and proficiency rates—of K‐12 students in math and reading from studies published between 2001 and 2016.

#### What is the aim of this review?

This systematic review synthesizes the findings from 30 studies that compared the performance of students at schools using single‐track year‐round calendars to the performance of students at schools using a traditional calendar.

#### 1.2.1 What studies are included?

This review includes studies that compare achievement in single‐track year‐round schools to achievement in traditional‐calendar schools. Of a total of 39 studies on the topic, nine reported outcomes in a way that could not be combined with the 30 that this review focuses on. The studies were from 2001 to 2016 and were all of K‐12 schooling in the United States, but varied in school characteristics (state, size, percent minority, percent low‐income). None of the studies used an experimental design (random assignment); studies were about evenly split between (a) comparing one school to another that is very similar, (b) comparing one school to a nearby school, and (c) comparing students at a single school before versus after a switch to a year‐round calendar.

### 1.3 What are the main findings of this review?

#### 1.3.1 Is academic achievement higher at year‐round schools?

Average student achievement was higher in both reading and math at single‐track year‐round schools. Compared to a prior meta‐analysis of summer learning loss which found that students typically forget the equivalent of 1 month of learning over the summer, this review found the gain from YRE to be slightly more than this in reading and a slightly less in math. Proficiency rates were not higher in either subject; possible reasons for this are discussed in the review.

#### 1.3.2 Do some students benefit more from YRE?

For the most part, no. Low‐income and minority students do not see greater benefit from YRE than average students in either reading or math. Elementary and middle school students show about the same gain in reading. However, we find that middle school students’ achievement in math increases more than elementary school students’ from the year‐round calendar. Because none of the included studies were experiments (and therefore factors other than duration of summer break may have been distributed non‐randomly), the certainty of these findings for smaller groups of students is lower.

#### 1.3.3 Do some year‐round calendars help students more than others?

Tentatively, yes: the schools that shortened summers to the fewest weeks had the largest effect on student achievement in both math and reading.

### 1.4 What do the findings of this review mean?

Single‐track YRE appears to have a benefit to student achievement that is equivalent in size to about a month of learning; this is similar in size to some ways of calculating the learning loss students experience over the traditional 10‐week summer break. In examining smaller subsets of data, which weakens the reliability of our analyses, the authors did not find YRE to be more helpful for low‐income or minority students than for the average student, but do find that YRE might have a larger effect for middle school students than elementary school students in math. Schools that shortened summer to the fewest weeks of vacation showed the greatest gain in student achievement, but the (non‐experimental) design of the studies examined preclude us from interpreting this relationship as causal. This might indicate that schools could expect an improved student achievement gain equivalent to 1 month of learning from a year‐round calendar, with a larger improvement from shortening the summer break to 4–6 weeks in length than from shortening the summer break to 7–8 weeks.

### 1.5 How up‐to‐date is this review?

The review authors searched for studies up to 2016, with electronic searches conducted in July and August 2017.

## 2 BACKGROUND

### 2.1 The problem, condition, or issue

#### 2.1.1 Summer learning loss

Summer learning loss is a prominent concern in academic and public discussions of education. Summer learning loss refers to the fact that students forget material and show measurably decreased competency over the period from the end of one school year in the spring to the beginning of the following school year in the fall. Concerns focus on not only what students forget over summer vacation, but also on the instructional time that must be spent reviewing previously taught material at the beginning of each school year. Overall, summer learning loss is worse in math than in reading (Cooper, Nye, Charlton, Lindsay, & Greathouse, 1996), likely because students read but do not do math during the summer. Cooper et al.’s (1996) meta‐analytic estimate was that achievement declines by approximately 1 month of learning (0.16 *SDs* in math and 0.11 in reading) during summer.

Longstanding research has shown that summer learning loss appears to be worse for historically disadvantaged students. Research has documented that low‐income students lose ground to higher‐socioeconomic status (SES) students during summer months when they cannot access school resources (Burkam, Ready, Lee, & LoGerfo, 2004; Entwisle, Alexander, & Olson, 2001). The magnitude of this loss relative to their more‐advantaged peers is substantial: low‐income students lose as much as 3 months of learning in reading over the summer (Von Drehle, 2010). In total, summer learning loss among low‐income students may account for as much as two‐thirds of the income‐based achievement gap (Alexander, Entwisle, & Olson, 2007). However, more‐recent analysis calls into question whether the difference in summer learning loss by income is robust to alternative research specifications (von Hippel, 2019; von Hippel and Hamrock, 2019) and even to analyses based on different standardized tests (von Hippel, Workman, & Downey, 2018). This complicates our understanding of the relative extent to which summer learning loss is evenly distributed across students or concentrated among low‐income and racial minority students.

The losses for historically disadvantaged students, documented in the earlier studies, align with research on differences in summer resources and opportunities. Low‐income students typically attend lower‐performing schools than their wealthier counterparts, but the resource differential in summer may be even greater (Downey, von Hippel, & Broh, 2004). During summer, less affluent children watch more television, converse less with parents, and have less daily parental involvement in general than do wealthier students (Gershenson, 2013). Wealthier students, in contrast, are more likely to engage in stimulating activities like taking lessons, visiting libraries, and attending museums than are less affluent students (Alexander et al., 2007).

### 2.2 The intervention

#### 2.2.1 Single‐track YRE

YRE is seen as a way to combat summer learning loss by shortening or eliminating the long summer vacation. YRE refers to the policy intervention of shortening summer break (and increasing the frequency and/or length of shorter breaks during the school year) to distribute instructional time more evenly throughout the year while retaining the standard 180 instructional days. The National Association for Year‐Round Education (NAYRE) defines YRE by saying that it provides “more continuous learning by breaking up the long summer vacation into shorter, more frequent vacations throughout the year…The year‐round calendar is organized into instructional periods and vacation weeks that are more evenly balanced across 12 months than the traditional school calendar” (NAYRE). One common calendar example alternates 45 instructional days (9 weeks) with 10 days (2 weeks) of break; this allocation of time is called a 45‐10 calendar, and results in a summer vacation of around 6 weeks instead of 10 or more.

YRE is sometimes conflated with other calendar and instructional reforms, so it is important to delineate how it is distinct from seemingly similar policies. YRE is distinct from a reform that is typically called extended year, which consists of adding days to the standard American school year of 180 days. YRE also does not refer to after‐school programming, tutoring, summer school for remediation, other summer programming, or lengthening the number of instructional hours in each school day. It refers *exclusively* to reallocating the 180 instructional days more evenly throughout the year.

Two distinct forms of YRE are commonly used but for different reasons. Single‐track YRE, in which all students are on the same schedule, is commonly a policy response to summer learning loss and is intended to improve student learning and achievement. In multitrack YRE, students are on multiple different calendars (typically four or five) so that a share of students are on break at all times (e.g., 20% of students on break and 80% of students in class in each week). Multitrack YRE is often a response to overcrowding as it increases the capacity of a school building without the cost of building new classrooms and other facilities. Because multitrack YRE is framed at addressing an issue other than summer learning loss, this review examines only the topic of single‐track YRE.

Single‐track YRE calendars themselves can vary on two important axes. Single‐track YRE can be implemented in a variety of calendar structures—whether a calendar has 30 days of instruction followed by 5 of vacation (called 30‐5), 45 days of instruction followed by 10 of vacation (45‐10), 45‐15, 60‐20, or another alternative—which could moderate the impact of the calendar type on student achievement. Single‐track YRE calendars can also differ in the duration of their summer vacation. Schools shorten their summer from the traditional 10 weeks to lengths ranging from 4 to 8 weeks; given the concern about summer learning loss, it would not be surprising for those lengths to moderate the effectiveness of single‐track YRE.

Year‐round calendars have become more common across the United States in recent years. According to Skinner (2014), from 2000 to 2012 the number of schools operating on a year‐round calendar increased from 3059 to 3700, representing 4.1% of all public schools in the U.S. in the 2011–2012 school year. The adoption of YRE also varies regionally and by school type. Schools in the South account for 40.5% of those that use a year‐round calendar, the largest share of any region, with the West containing 24.3% of the country’s year‐round schools and the northeast and midwest each accounting for 16.2% of U.S. schools operating on a year‐round schedule (Skinner, 2014). This growth in the adoption of YRE points to the importance and timeliness of research examining the impact of this reform on student achievement.

### 2.3 How the intervention might work

The logic of YRE is fairly simple: by redistributing the school calendar to create shorter breaks in which there are fewer *consecutive* weeks for students to forget material, the degree of learning loss during the summer will be lessened, which in turn means that students will need less review after breaks and allow teachers to cover more material over the course of an academic year. The thinking of advocates is that the more‐frequent short breaks (e.g., of 2 weeks, in a 45‐10 calendar structure) are not long enough to engender learning loss in the same way that lengthy summer vacations do. This reveals an important assumption on the part of YRE advocates, which is that learning loss is a nonlinear function of the rate at which students forget what they have learned and time. If the relationship between time off school and learning loss are indeed linear, then YRE would not be able to counter summer learning loss because altering the calendar would not change the total amount of time that students spend in and out of school. Students would then forget a smaller amount during each break, but the total learning loss would still sum to the same annual total as on a traditional calendar. If, on the other hand, the relationship between time spent outside of school and learning loss is nonlinear, such that the degree of learning loss is minor over short periods of time but becomes more severe over longer periods, then altering the school calendar to create shorter breaks should decrease overall learning loss. If correct, distributing vacations and schooling more evenly throughout the year would allow for students’ year‐over‐year academic progress to increase with no additional days of teaching.

### 2.4 Why it is important to do the review

Two prior meta‐analyses have examined the effect of YRE’s on academic achievement, primarily with subjects merged into a single composite academic outcome. Kneese (1996) included both studies with comparison groups and pre/post studies, and found a positive effect on achievement varying from +0.11 to +0.2 *SDs* depending on the exact model and analysis used. Kneese also stated that single‐track calendars appeared to have a larger effect than multitrack calendars. Cooper et al. (2003) included only studies with comparison groups, and found an overall effect size of +0.06, but that this increased to +0.11 when restricted to studies that used statistical or matching controls. Cooper et al. (2003) disaggregated by calendar type, and in their fixed‐effects unadjusted analyses found that, although multitrack YRE had an effect size of −0.01 (±0.05), single‐track YRE had an effect size of +0.16.

These prior reviews provided important information on how YRE overall relates to student learning. However, the Cooper et al. (2003) study included research through 2000. Since 2001, in the NCLB and post‐NCLB era, schooling in United States has experienced a broad array of shifts and interventions. These may have introduced systemic differences in the effect of YRE. Perhaps more importantly, the prior reviews focused on YRE overall, and only examined single‐track YRE as a whole (that is, combined achievement in reading and math) compared to multitrack YRE as a whole. By focusing only on single‐track YRE, we will be able not just to arrive at an overall effect size estimate for both math and reading, but also to begin observing both qualities that make single‐track YRE more effective and student populations for whom it is more effective.

The findings from this meta‐analysis can provide guidance to policymakers about the efficacy of single‐track YRE as an intervention to increase student achievement, and for which schools and students it is most likely to be effective.

## 3 OBJECTIVES

### 3.1 Understanding effects of single‐track YRE and its characteristics

#### 3.1.1 Research questions

Guided by prior research, this meta‐analysis examines single‐track YRE only. The main objective is to identify, across studies published in the post‐NCLB era, how single‐track YRE affects student achievement. Along with this, we investigate the effect of YRE on different subgroups of students. The summer learning loss literature shows that historically disadvantaged students fall further behind their advantaged peers over the summer. This disparity points to the possibility that the effect size of YRE, which derives in part from mitigating summer learning loss, may differ for subgroups of students. Third, given the assumption of YRE advocates that learning loss is a nonlinear function of time, we also examine the relationship between the effect single‐track YRE and the structure of the calendar implemented. We operationalize these objectives in the following research questions:

1. What is the estimated effect of single‐track YRE for math achievement and for reading achievement?
2. What is the effect size (of math and reading achievement) for only low‐income students and for only minority students?
3. What is the relationship between characteristics of YRE (calendar structure, duration of the longest remaining break) and the effect size estimate?

## 4 METHODS

### 4.1 Criteria for considering studies for this review

#### 4.1.1 Types of studies

As is commonly the case in education research, we did not encounter any experimental studies. Much research in this area is simply mean achievement comparisons at schools with similar demographic characteristics. In order to avoid excessively restricting the size of our final sample, we included studies that use any approach to comparing academic achievement at traditional calendar schools versus single‐track year‐round schools (the protocol for this review is available at Fitzpatrick & Burns, 2017). This includes single‐track year‐round schools compared with a comparison group based on: matched school‐level characteristics, matched student‐level characteristics, and geographic proximity (e.g., within a small county). We excluded any studies that do not include achievement data. Many analyses are only of differences in average achievement (at one school or at multiple schools; sometimes using student‐level data and sometimes using school‐level data), so we include these mean comparison data. We also include multivariate observational studies, which for this meta‐analysis typically means ordinary least squares regression.

We apply an exclusion criterion that studies must include a comparison group. Pre/post comparisons are not accepted in Campbell review so we do not include studies that use a comparison of the performance of a single group of students both on a traditional calendar and (in a subsequent year) on a year‐round calendar. However, a subset of YRE evaluations use what we call *cohort* designs (e.g., comparing the performance of students in Cohort 1, who were on a traditional calendar, to students in Cohort 2, who were on a year‐round calendar that was newly implemented, where Cohorts 1 and 2 are all enrolled students (in a given grade) at the same school). Scholars disagree about the strength of cohort designs relative to matched designs (see, inter alia, Cheng et al., 2016).^1^ Because of that tension, we conduct a sensitivity analysis of how including cohort comparison studies shifts the estimated average effect size. Given how common cohort comparisons are and the proportion of available effect sizes that they represent, though, it would be inappropriate to exclude them entirely. We therefore consider comparing the performance of a group of students on YRE, to the students in that school and grade during prior years (and on a traditional calendar), as having a comparison group

#### 4.1.2 Types of participants

Studies must be of K‐12 schooling (students). Both early childhood education and college have enough differences in policy and practice from K‐12 that a cross‐level merged effect would not be appropriate. The restriction to K‐12 schooling will allow for effect estimates to be for primary and secondary education, which are commonly grouped, without including studies examining modified school calendars in early childhood education, preschool, or college. Additionally, we consider studies of whole schools or of only regular‐education students (who are in some cases the only students for whom achievement data are available), but not any studies of special education students. We initially planned to separately estimate effects for U.S.‐only results and international results.^2^ However, all studies included in the final sample were in the United States or United States territories.

#### 4.1.3 Types of interventions

Year‐round calendars are not all the same. The most important distinction in type is whether a calendar is single‐ or multitrack. On a single‐track calendar, all students and teachers are on the same schedule (track). The school building either has all students present or none present on each day, and the building only has students in it for 180 days per year. Single‐track YRE is typically framed as an academic reform to improve student achievement. In contrast, multitrack YRE is typically implemented in response to overcrowding when there is no funding available for additional classroom space. On a multitrack calendar, some of the students (e.g., 25%) are on vacation at any given time, while the other students (in this example, 75%) are in school. The tracks rotate through their time in school and on vacation, which allows a school with capacity for 900 students to serve 1,200 students on a rotating basis.

Multitrack calendars introduce disadvantages that are unique to having multiple tracks. Administrators and support staff need to serve all tracks, and may bear a heavier workload than on a traditional calendar (Ballinger & Kneese, 2006). Siblings can be on separate tracks, meaning that they have vacation at different times; faculty meetings are difficult to schedule because some teachers are on vacation at most times. Teachers have to share classrooms or may have to use a mobile cart to teach in multiple classes. Because the school is in use for at least some students during nearly all weeks, it can be a challenge to schedule renovations or other facilities work.

Individual studies that examined both single‐ and multitrack YRE have found that single‐track schools showed larger performance gains (e.g., Turk‐Bicakci, 2005; White & Cantrell, 2001). Conversely, the effect of multitrack YRE may actually be negative (Graves, 2010; Graves, McMullen, & Rouse, 2013). In both the Kneese (1996) and Cooper et al. (2003) meta‐analyses, the authors found a larger treatment effect for single‐track than multitrack YRE. Estimating the effect of grouped single‐ and multitrack YRE as a single treatment of “year‐round education” would require ignoring the important guidance provided by prior research findings. As a result, the current study excludes multitrack YRE—as an overcrowding/financial intervention previously shown not to contribute to student achievement—and focuses only on single‐track YRE because it is an academic intervention previously shown to have a modest but significant positive effect.

#### 4.1.4 Detailed challenges of multitrack schools

One set of problems stems from the fact that a fraction of classes are on break at all times. Because there are multiple schedules within a school, siblings can end up on different tracks (Glines, 1997; Shields & Oberg, 1999). If a family goes on a trip during one student’s vacation, one sibling might be pulled out of class. At any given time, multitrack schools have classes on break, and teachers of those classes are typically unavailable. This can impede communication within the school (Alkin, Atwood, Baker, Doby, & Doherty, 1983; Rodgers, 1993). The lack of communication can lead to disunity among teachers and staff (Severson, 1997; Shields, 1996). The split schedule can also have negative interactions with standardized testing (California Department of Education, n.d.). In an extreme example, one track of students may return from a multiweek break just a few days before annual testing, which may create inequities in test preparation across tracks (Helfand, 2000).

In all or nearly all weeks of the year, at least some students are attending classes in a multitrack school. This near‐constant use of the school creates a second set of problems. The school must operate more days, increasing demands on support staff like custodians and teacher aids. Administrators are needed year‐round, as they must work when any track is in operation, substantially increasing fatigue among administrators (Mutchler, 1993). Continuous use of the school building also impedes any large facilities work (Mussatti, 1981) and in some cases makes routine maintenance and repair more difficult (White, 1993). If teachers supplement their income by assisting on a track they do not teach, they also lose the option of engaging in lesson planning between school years (St. Gerard, 2007). Given that some teachers are not working at nearly all times, it is also difficult to schedule staff‐wide professional development.

A third set of problems result from the use of a multitrack relative to a single‐track schedule. Each classroom has to serve multiple tracks, so teachers share classrooms (Dixon, 2011). In some cases teachers have to set up and take down their classroom every few weeks; in others, teachers have mobile carts to move between classrooms. Since faculty are on differing schedules, creating a sense of community can be exceptionally difficult (Rakoff, 2002). Either approach interferes with teacher performance. Of significant concern, Mitchell and Mitchell (2005) found substantial racial segregation between tracks. Parental requests for specific tracks can contribute to uneven distributions by SES and race (McNamara, 1981; Sparks, 2002). In some multitrack schools, English Language Learners are unevenly distributed across tracks as well (Brekke, 1986). Multitrack calendars can also worsen the effects of academic tracking: in addition to not being in classes with students of differing academic abilities, students may not be in the school building on the same schedule as students of differing ability.

#### 4.1.5 Types of outcome measures

##### Primary outcomes

The outcomes for this meta‐analysis will be (a) math achievement scores and (b) reading achievement scores, measured both by mean scores (including both mean scores and mean percentile scores) and by percent proficiency or other dichotomous outcomes.

##### Secondary outcomes

Supplementary analyses examine growth as an outcome (instead of only single‐year achievement scores). Growth scores are not consistently available in studies included in the final sample, so growth analyses are suggestive rather than comprehensive.

#### 4.1.6 Duration of follow‐up

We consider only studies that examined outcomes while students were still attending the year‐round school. This restriction excluded only a single dissertation, which examined the high school achievement of students who had attended a year‐round elementary school.

#### 4.1.7 Types of settings

We examine studies in which single‐track YRE was the only schedule‐based intervention. Studies cannot be evaluations of extended instructional time (e.g., lengthened school day or additional instructional days). It is not infrequent for schools or school districts to make multiple changes at once. However, it would not be possible to identify what share of a change in student performance was due to a year‐round calendar (i.e., the elimination of summer learning loss) and what share was due to additional days of instruction. We therefore only include studies of schools on year‐round calendars without extended instructional time or other simultaneous calendar reforms.

### 4.2 Search methods for identification of studies

#### 4.2.1 Electronic searches

Our general/starting‐point search terms for this meta‐analysis include those used by Cooper et al. (2003), augmented by terms used in pertinent research published after that meta‐analysis. The basic form of the search terms is: “year‐round school*” or “year‐round education” or (school AND (“alternative calendar” or “modified calendar” or “balanced calendar”) or (“year‐round calendar” AND school). We modified the precise terms, phrases, and Boolean operators to take advantage of the search features, index terms identified in the resource’s thesaurus, and tools within each of 22 specific search/retrieval resource. Searches were restricted to studies dated 2001–2016, to avoid duplicative inclusion of studies that were in the Cooper et al. (2003) work. As searching was conducted, records were saved in Excel for each search result, which allows for clear indication of which results were found by each database/tool (for both sources found in multiple sources, and for unique results). Additionally, we recorded the reason(s) that studies failed to meet study criteria. Electronic databases searched were:

- ERIC
- PsycARTICLES
- PsycEXTRA
- PsychINFO
- ProQuest Research Library
- ProQuest Dissertations & Theses Global
- Dissertations & Theses @ CIC Institutions
- Education Administration Abstracts
- Education Full Text
- Social Sciences Citation Index
- Sociological Abstracts
- PolicyFile
- International Bibliography of the Social Sciences
- Periodicals Index Online
- EconLit
- Sociology Database
- PRISMA
- Social Services Abstracts
- PAIS International
- Google Scholar
- Google [for identifying grey literature; intending to review the first 400 results]
- Web of Science

We include a database search log in an online appendix to this review. This log contains, for each database that was searched, the terms, phrases, and Boolean operators that were used to identify relevant studies; fields that were searched; and restrictions or filters that were used. The log also includes comments on the search strategy used for each database to describe any database‐specific procedures that were used to identify studies. Finally, the log indicates the number of records that were retrieved from each database along with the number of full‐text studies that were downloaded from this pool. At all steps, our search process adhered to best practices in research synthesis as outlined by the Campbell Collaboration (Kugley et al., 2017).

In addition to searching databases, our research synthesis protocol included footnote chasing in two directions. Using the “cited by” feature on both ProQuest and Google Scholar, we examined all publicly available works that cited the Cooper et al. (2003) meta‐analysis or any study added to the final sample (sometimes called “cited reference searching”). Additionally, for each study that met the selection criteria, all footnotes were reviewed and any studies that were not already part of the sample were added from this traditional footnote chasing.

Finally, we conducted searches or reviewed the titles of all reports (depending on number of reports and available search interface on individual, e.g., corporate, websites) to identify additional grey literature from pertinent websites. Those sites include the more than 50 (excluding higher education‐specific resources) listed in the Campbell information retrieval guide (Hammerstrøm, Wade, & Jørgensen, 2010).

### 4.3 Data collection and analysis

#### 4.3.1 Selection of studies

The results from the initial search included a large number of works that were not actually studies warranting inclusion in this meta‐analysis. Four selection criteria, adapted from those used by Cooper et al. (2003), were applied to identify those that were viable evaluations of the effect of YRE in the United States:

- Studies cannot be evaluations of extended instructional time (e.g., lengthened school day or additional instructional days).
- Studies must include quantitative achievement data.
- Studies must include a comparison group.
- Studies must be of K‐12 schooling in the United States

**Figure 1:**
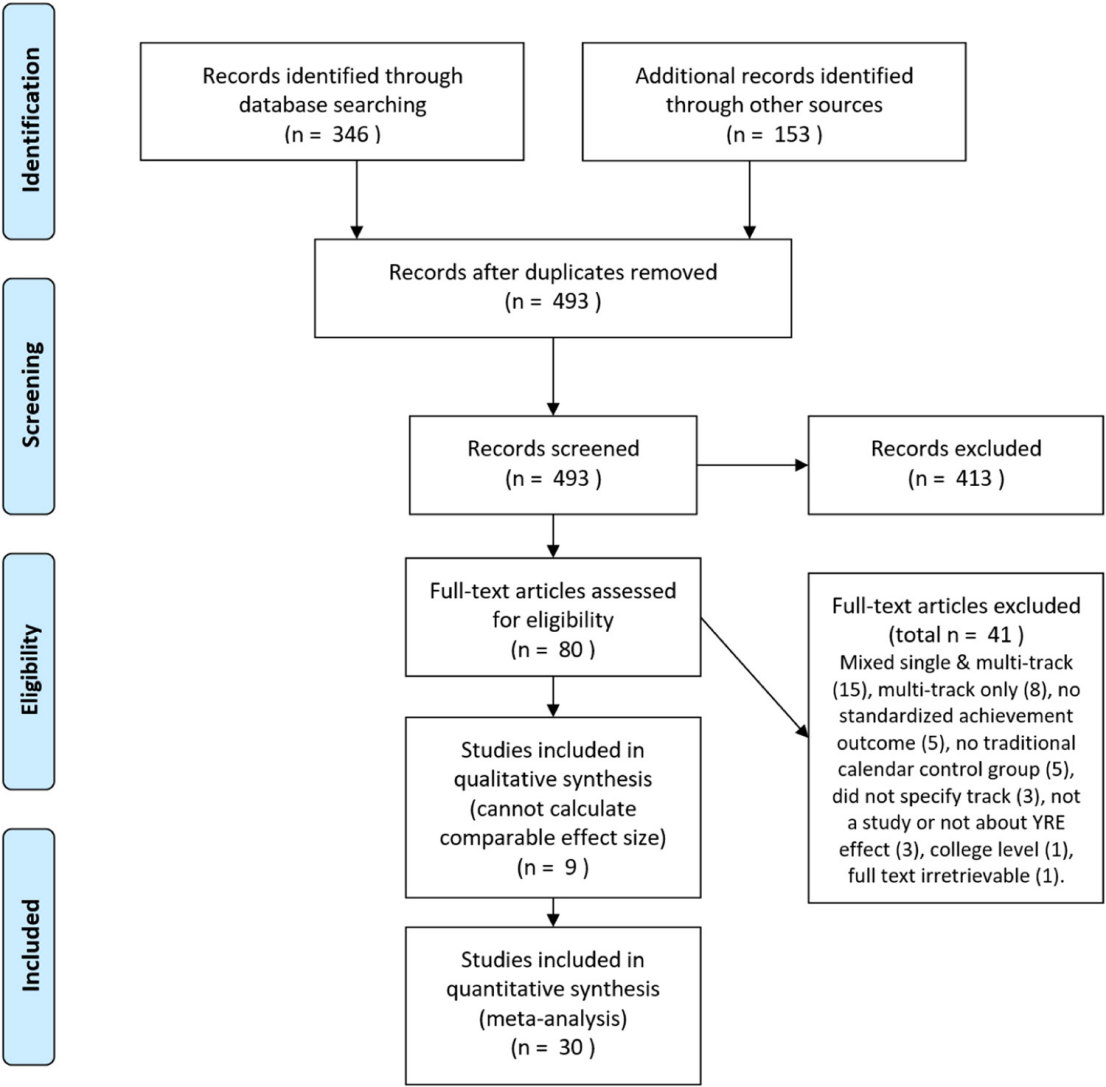
Search process flow diagram, adapted from Moher et al. (2009) [Color figure can be viewed at wileyonlinelibrary.com]

Figure 1 shows the flow of included documents from initial search through final sample. One elective restriction was applied deliberately in order to more accurately address a narrower research question, despite the resulting limited sample size. As noted above, only studies of single‐track YRE were included. Studies of multitrack YRE were excluded, as were studies that mixed single‐ and multitrack YRE and studies that did not specify the calendar type. This analytic restriction eliminated a large percentage of the initial sample: 26 studies were excluded for one of those three reasons. The exclusion was applied because prior work indicates not just that the two calendars are introduced for different reasons and suffer different disadvantages, but furthermore that multitrack YRE may have no treatment effect, whereas single‐track YRE has been found to have a positive effect. Some studies also lacked the information necessary to calculate an effect size and were excluded for that reason.

#### 4.3.2 Data extraction and management

##### Student outcomes

We extracted the student outcome data needed for calculating the effect size(s) from each study. In most cases this was mean score, *SD*, and sample size (*N*) for the treatment and control groups, or *N* and percent proficient. When necessary, we extracted data from other analyses such as *F* tests and analysis of variance (ANOVA). When multiple estimates were provided instead of a single overall treatment/control estimate (e.g., values for three grades or over 3 different years) we extracted the data for multiple effect size estimates from that study. In addition to full‐school statistics, where available, we extracted the data necessary for calculating effect sizes for subgroups of the full sample: for low‐SES students only (24 estimates from 10 studies) and for racial minority^3^ students only (35 estimates from 11 studies). Note that our subgroup analyses include the full‐study estimates for the few studies whose treated students were 100% eligible for free or reduced price lunch (FRPL) or were 100% minority.

##### Calendar characteristics of interest

To consider our second research question, we recorded two independent variables of interest: calendar structure and the duration of summer break. Single‐track YRE calendars can differ from each other on two important axes: calendar structure and the length of summer break. Single‐track YRE can be implemented in a variety of calendar structures—whether a calendar has 30 days of instruction followed by 5 of vacation (called 30‐5), 45 days of instruction followed by 10 of vacation (45‐10), 45‐15, 60‐20, or another alternative—which could moderate the impact of the calendar type on student achievement. Unfortunately, calendar structure was inconsistently reported. Of studies in the final sample, only 12 (40%) reported a single calendar structure implemented in all treatment schools. Another six (21%) reported the combined performance of multiple schools following different calendar structures. Though 11 (38%) did not provide calendar structure information, we contacted authors and were able to add structure information for eight of them. Table 1 thus shows a calendar structure for 20 (67%) studies, revealing that the 45‐10 structure was recorded twice as often as any other structure.

**Table 1:** Characteristics of studies in final sample

Study author and year	Math ES	95% CI, M^g^	Reading ES	95% CI, R^g^	YRE students (*N*)	State	Calendar structure	Weeks of summer	Grade level
*Continuous outcome of mean achievement score*									
Abakwue (2011)	+0.10		+0.36		120	TN			8
Carl (2009)	+0.69		+0.35		726	WI	10‐day breaks	~4	3–6
Cary (2006)	+0.07		+0.20		466	VA			3, 5
Coopersmith (2011)	+0.21	−0.24, 0.66	+0.13	0, 0.26	7,148	TX	45‐15^a^	4–6^a^	6–8
Crow (2009), Crow and Johnson (2010)^c^	−0.14	−0.31, 0.03	+0.00	−0.20, 0.21	163^b^	TX	45‐10^a^	8^a^	3–5
D’Alois (2005)	+0.13	−1.57, 1.83	+0.02	−4.48, 4.52	167	VA	45‐10	4	3, 5
Fritts‐Scott (2005)	+0.05	−4.84, 4.93	−0.09	−1.76, 1.59	451	AR	Mixed	8	1–3
Graves (2009, 2010)	−0.04	−0.33, 0.24	+0.06	−1.51, 1.62	~17,000	CA	Mixed	Mixed	Avg. 3.6
Lindsay‐Brown (2010)	−0.04	−2.81, 2.74	−0.16	−2.44, 2.12	113	SC	45‐15	6	4
Malicsi (2003)	−0.14	−3.01, 2.74	+0.52	−0.74, 1.78	1,099	Guam	45‐15		1, 3, 5
Marks (2006)	+0.05	−0.18, 0.28	+0.10	−2.12, 2.31	695	TN	45‐10	8	6
McLean (2002)	+0.35	−0.26, 0.97	+0.15	−0.16, 0.45	71	OH	45‐15	5	5–8, 11
McMillan (2005)	+0.16	0.05, 0.27	+0.14	0.10, 0.19	219	TN	45‐10^a^	7	3–5
Merrill (2012)			+0.11	−2.33, 2.55	42	IL	45‐10^a^	6	5
Moore (2002), Moore and Verstegen (2004)^d^, ^e^	+0.28	−1.25, 1.82	0.03	−2.64, 2.71	64	VA	Not standard	~6	3–4
Ramos (2006, 2011)^e^	+0.29	−3.17, 3.75	+0.48	−2.93, 3.89	74	CA/ID/IA	45‐15	“~6”^a^	5
Sexton (2003)^e^	+0.27	−9.04, 9.49	+0.08	−9.87, 10.02	87	VA			8
Thomas (2002)	+0.28	−0.47, 1.04	+0.31	−0.41, 1.04	446	TX	3/4 30‐5		10
Trent (2007)	+0.16	0.05, 0.27	+0.14	0.10, 0.19	330	TN	45‐10^a^	7	6–8
Varner (2003)	+0.01	−1.25, 1.26	+0.48	0.05, 0.92	146		45‐15^a^	“slightly over 8”	3
Wilmore‐Dafonte (2013)^c^	0.06^a^	0.02, 0.11	+0.08^a^	0.02, 0.14	11,608	TX	Mixed	Mixed	5
*Dichotomous outcome (percent proficient, percent passing, etc.); ES in odds ratio*									
Beazley (2001)	1.21	0.17, 8.59	1.02	0.48, 2.18	1,307	AZ	Atypical	6^a^	9–12
Carl (2009)	0.79	0.68, 0.92	0.83	0.77, 0.91	3,228	WI	10‐day breaks	~4	3–6
D’Alois (2005)	1.54	0.05, 48	1.37	0, 419	297	VA	45‐10	4	3, 5
Evans (2007)^f^	2.15		3.37		17^b^	IN			3
Ferguson (2001)	3.96		0.54		67	VA	45‐10		5
Helton (2001)	0.98	0.79, 1.21	0.91	0.74, 1.12	23^b^	FL		“~5”	4–5
Kellems (2006), Oppel (2007)^f^	1.34	1.13, 1.59	1.25	0.14, 10.88	656	IN	45‐10^a^		3, 6, 8, 10
Mitchell‐Hoefer (2010)	1.34	0.30, 5.90	0.95	0.21, 4.28	704	SC	45‐10^a^		3–5
Schumacher (2015)	1.09		1.12		444	NE	Atypical	5	3–5
Thigpen (2004)	0.55	0.19, 1.64	0.59	0.25, 1.38	65	MS	45‐15		2–5
Winkelmann (2010)	0.88		0.99		40^b^	IL	45‐15 most common	6	3

*Note:* Data extracted from primary study documents.

Abbreviations: CI, confidence interval.

^a^ I am indebted to the authors who shared additional, unpublished data for inclusion in this meta‐analysis.

^b^ The sample for this study is buildings, not students (student‐level results were not provided), so its results may be under‐weighted in analyses.

^c^ Both studies include fifth graders in Texas in 2006–2008, so it is possible that these results include the same students (both studies anonymized the schools analyzed). This would involve a maximum of 164 students’ results, so it should not bias the results in a significant fashion even if those students are included twice.

^d^ Parental sign‐up for the year‐round school was voluntary.

^e^ Single track school colocated with a traditional calendar school.

^f^ The third graders in Kellems (2006) and Oppel (2007) represent 2 of the 58 estimates in the Evans (2007) study.

^g^ For studies with multiple estimates, CI provided is for the weighted average of within‐study estimates; for studies with a single comparison, CI provided is for Hedge’s *g*. Figures would not be directly comparable for studies with a single dichotomous outcome comparison (Evans, 2007; Ferguson, 2001; Schumacher, 2015; Winkelmann, 2010) or studies for which Hedges’ *g* was calculated based on a figure without *SD* information, such as an *F* test (Abakwue, 2011; Carl, 2009; Cary, 2006).

Single‐track YRE calendars can also differ in the length of their summer vacation. Schools shorten their summer from the traditional 10 weeks to lengths ranging from 4 to 8 weeks. Given that single‐track YRE is predicated on diminishing summer learning loss, it would not be surprising for those lengths to moderate the effectiveness of single‐track YRE. The consistency with which studies reported the year’s longest break resembled that of calendar structure, with 14 (47%) reporting a break length and another 2 (7%) reporting the combined performance of multiple schools with breaks of different lengths. Again, we contacted authors and gained supplementary un‐published data from 4 (14% of) authors about the length of summer break, but for 10 studies (34%) no data were available. The studied schools with available summer length data show large variation in that length: one as short as 4 weeks, three at 5 weeks, six at 6 weeks, two at 7 weeks, and four at 8 weeks long.

##### Study, school, and sample characteristics

For each study, we recorded standard information on the study and report itself. This included the report author, year of publication or release, published/unpublished status, and the matching protocol used to identify the comparison school(s). For the treatment schools examined, this included the state in which the schools were located, years of student testing data included, and the type of score used for the outcome measurement. We also recorded sample/student characteristics associated with each estimate. For studies that separately reported the outcomes for multiple student groups, we recorded these data separately for each estimate within those studies. We coded the grade range of the students tested, a value for school type (elementary [K‐5], middle [6–8], or high [9–12] school), the percent of treatment‐group students that were Hispanic or African‐American (subsequently referred to as “minority”), and the percent of treatment‐group students that were eligible for FRPL or otherwise were designated low‐income.

#### 4.3.3 Assessment of risk of bias in included studies

Examining the studies included in this meta‐analysis revealed two potential sources of bias in our results: publication bias and bias arising from the internal validity of included studies. While publication bias is a concern in any meta‐analysis, we argue that the risk of publication bias in this review is low because the majority of studies in the final sample are unpublished dissertations and reports. While this does not mean that publication bias can be definitively ruled out, we are confident that the present meta‐analysis includes all the relevant and available research on YRE from 2001 to 2016. However, bias stemming from identification strategy is of greater concern because the designs and/or analytical strategies employed by studies retained in this meta‐analysis may pose a threat to their internal validity. Reviewing the studies retained for this meta‐analysis, we observe three different strategies used for identifying comparison groups: geographic proximity, student cohorts, and using student and/or school characteristics to identify a comparison group. While there are strengths and weaknesses to each approach, the degree to which geographically selected comparison cases make for a valid counterfactual is unclear. On one hand, selecting proximate schools and/or districts for comparison could meaningfully account for a range of contextual factors. On the other, student characteristics and achievement may vary considerably over even small spatial differences which, if not accounted for in a study’s analytical strategy, may bias estimated effects, though it is difficult to determine the direction and magnitude of such bias.

To investigate this issue, in the results, we conduct separate analyses of those studies that simply used geographic proximity to identify a comparison group, studies that used a cohort design to assess how a particular school’s (or how particular schools’) performance changed after conversion to a year‐round calendar, and studies that used a matching protocol. Comparing these results, we find that the estimated effect sizes are consistently positive, but that the magnitude of these effect sizes vary significantly based on identification strategy. Specifically, analyses restricted to studies that use geographic proximity obtain larger effect sizes and cohort designs produce more varied effect sizes than do matching protocols. As a result, the overall effect sizes we observe may be biased, though the direction of this bias is unclear.

Formal tools for assessing bias in meta‐analysis were developed based on meta‐analyses of randomized controlled trials (RCTs) with, for example, differing approaches to randomization or single‐ versus double‐blind treatment assignment. For instance, the Cochrane Collaboration risk of bias tool (Higgins et al., 2016; Higgins & Green, 2011; Higgins et al., 2011) is designed for use with RCTs. The Cochrane Risk of Bias Assessment Tool for Non‐Randomized Studies (ACROBAT‐NRSI; Sterne, Higgins, & Reeves, 2014), was designed for use with studies applying quasiexperimental designs, as were What Works Clearinghouse (WWC) tools for non‐RCT studies. No quasiexperimental studies remain in our final sample.

Even the more generalist EPOC risk of bias tool includes a number of criteria (sequence generation, allocation concealment, blinding of outcome assessment, and protection against contamination) that are misaligned with observational studies of school‐level policy change (see Cochrane Collaboration, 2017a). There are no observable reasons to predict differing levels of incomplete outcome data reporting or selective outcome reporting across these studies, which all state that they used all available general‐education achievement data in the treatment and comparison schools. From the EPOC framework, the two potential areas for concern are baseline outcome equivalence and baseline characteristics equivalence. These are both issues of how (and how well) the comparison population was identified. In our findings (in Table 4 and discussed in “Risk of bias in included studies”) we therefore carefully consider how the three types of comparison populations used by studies in the final sample may have contributed bias, which is more aligned with a summary assessment of risk of bias (see Cochrane Collaboration, 2017b). We are unable to assess the direction or magnitude of bias resulting from baseline differences in characteristics. A treated group with higher baseline achievement could bias effect estimates upwards due to their academic ability; but a treated group with higher baseline achievement could bias effect estimates downward, if they are students who take advantage of summer opportunities that minimize summer learning loss. We are unable to specify the direction of bias introduced by the fact that our final sample consists of comparisons to prior year, matched schools, or nearby schools, but that limitation adds uncertainty to our meta‐analytic estimates.

The Cochrane Collaboration Risk Of Bias In Non‐Randomized Studies—of Interventions (ROBINS‐I) is has partial applicability to studies examining outcomes before and after a universally applied (within schools) policy change (Sterne et al., 2016). These concepts are most useful in the present case as applied to the *body of studies* rather than to individual studies. Preintervention considerations are where (across all studies of this topic) there is serious risk of bias. The schools and communities that decide to switch to YRE may have characteristics that interact with the effectiveness of the new calendar (as both are likely related to a community’s attitude toward education). This possible bias due to confounding is of greater concern than any other areas. Because the policy change occurred at the school level, individual students likely introduced little or no bias in the selection of participants into the study. Our selection criteria deliberately excluded studies with multiple intervention types, so our analyses have low risk of bias from the classification of interventions. Missing data and selective reporting are both discussed above. Because the same standardized tests were used in YRE and traditional calendar schools, (a) the studies in our final sample have relatively low risk of bias in outcome measurement, and (b) the bias introduced by standardized testing is common to education research.

#### 4.3.4 Measures of treatment effect

We used the data in each study in the final sample to calculate one or more effect sizes for math and for reading. For continuous outcomes we calculated Hedges’ *g*, which is the difference in outcome between the treatment and control groups divided by their pooled *SD*, with a correction for upward bias that Cohen’s *d* introduces for small samples (Borenstein, 2009).^4^ For dichotomous outcomes—percent proficient, percent passing, and so forth—we calculated and combined logged odds ratios (Fleiss & Berlin, 2009). Findings are presented in odds ratios, for ease of interpretation. The two types of outcome are analysed separately both to allow for interpretation of meta‐analytic estimates to remain close to the results of the original articles, and also because it would not be surprising for there to be a larger difference in means than in dichotomous outcomes.

Although dichotomous outcomes can be rescaled into estimates to be combined with Cohen’s *d* or Hedges’ *g*, doing so is an imperfect approach. For example, alternative calculations for rescaling dichotomous outcomes have different properties (Sánchez‐Meca, Marín‐Martínez, & Chacón‐Moscoso, 2003). Odds ratios are the consensus best‐available approach to dichotomous outcomes (see, inter alia, Haddock, Rindskopf, & Shadish, 1998; Olivier & Bell, 2013). However, estimates of odds ratios may be less valid than other effect size types (e.g., Cohen, 1983; Durlak, 2009; Hsu, 2004; Hunter & Schmidt, 2004) and are very sensitive to base rates (Ruscio, 2008). Furthermore, measurements of odds ratios are extremely sensitive to the cut‐points used (see, e.g., Chen, Cohen, & Chen, 2007; Cohen & Chen, 2009; Okada & Hoshino, 2017); given that benchmarks for proficiency differ across states and tests, this possible source of bias is particularly concerning in education. Despite this, rescaling is recommended (e.g., Polanin & Snilstveit, 2016) in cases where a few odds ratios join a majority of mean differences (e.g., 1:5 or 3:7) in a final sample. Because slightly over 1/3 of studies in our final sample report dichotomous outcomes, though, our sample includes enough for a separate analysis of those estimates as a group, rather than merging outcome measures with different statistical properties into a single, composite outcome. Furthermore, in the case of YRE specifically, there are also substantive reasons to think that the treatment might have different effects on the two outcome types. Given that YRE is intended to combat summer learning loss, which is concentrated among lower‐SES and often lower‐performing students, the effect of YRE might be to improve the mean achievement of below‐proficient students, but without shifting them to proficiency. Merging the two types of estimate into a single composite outcome would have methodological limitations and might lose distinctions in what is being measured, without providing sufficient benefit to offset these disadvantages.

The final sample in reading was 58 *g* and 31 odds ratio estimates from 30 studies; the final sample in math was 55 *g* and 29 odds ratio estimates from 29 studies. Notably, the final sample is predominantly studies of primary schooling (grades 3–8) and is mostly unpublished dissertations.^5^

#### 4.3.5 Unit of analysis issues

The final sample in this meta‐analysis included a small enough number of studies that it was straightforward to assess whether any covered the same state in the same years of testing. In such a case, the studies—for those that anonymized the results—could feasibly of the same students. Two studies, by Kellems and Oppel, are merged because they precisely duplicate the population: the same Indiana school system in the same year. Otherwise, <1% of records could feasibly be the same students.

##### Studies with dependent estimates and final meta‐analytic calculation

The structure of the data from our final sample complicated selecting a final model for estimating the average effect size for single‐track YRE. The effect sizes extracted from studies with multiple estimates were heterogeneous in their structure. Twelve studies reported one estimate, the remainder had more than one estimate, but not with a consistent hierarchical relationship. Several provided multiple grades of data for the same year, multiple years of data for the same grade, or reported multiple races for the same grade in multiple years. While those data structures do not create statistical dependencies in the estimates, three studies provided estimates following the same cohort of students (or multiple cohorts) for multiple years, which would have correlated errors among the repeated measures of the same students if all estimates were included in a weighted average. Common approaches to meta‐analytic calculations for studies with multiple effect size estimates were not appropriate for these data, but robust variance estimate (RVE) was.

Several typical techniques for resolving within‐study dependence are not suitable to the single‐track YRE effect sizes. It is common to calculate a simple or weighted average of multiple effects size estimates from a study in order to produce a single estimate for that study (used in 42.9% of meta‐analyses according to Ahn, Ames, & Myers, 2012). This aggregation approach, though, does not properly account for the correlation among those within‐study estimates (see Becker, Hedges, & Pigott, 2004; Gleser & Olkin 2009; Kim & Becker, 2010; Raudenbush, Becker & Kalaian, 1988). Multivariate meta‐analysis is the most common approach for addressing dependence among estimates (see Gleser & Olkin, 2009; Hedges & Olkin, 1985; Raudenbush, Becker & Kalaian, 1988), but it requires within‐study correlation statistics (Becker et al., 2004; Jackson, Riley, & White, 2011) which are not available for our final sample. Three‐level meta‐analysis may be able to account for hierarchically structured effect size estimates (Konstantopoulos, 2011), but there are insufficient estimates in this final sample for a three‐level model to be appropriate. Meta‐regression would also be mismatched without a larger sample of studies (Borenstein, Hedges, Higgins, & Rothstein, 2009).

Meta‐regression with RVE addresses precisely the data problem in the single‐track YRE dataset. RVE was developed to estimate meta‐regression coefficients in models with dependent effect sizes and properly account for those statistical dependencies when the structure of their dependence is unknown (Hedges, Tipton, & Johnson, 2010a, 2010b). In a test of possible ways to address dependence in effect sizes, RVE estimates were found to be consistent with other methods, and both the effect size and heterogeneity estimates were robust to variations in the intraclass correlation value *p* (Scammacca, Roberts, & Stuebing, 2014). RVE has been validated (Moeyaert et al., 2017) and is increasingly used to account for the dependence of multiple within‐study estimates in meta‐analyses in education (e.g., Clark, Tanner‐Smith, & Killingsworth, 2016; Conn, 2017; Dietrichson, Bøg, Filges & Klint Jørgensen, 2017; Gardella, Fisher, & Teurbe‐Tolon, 2017; Swanson et al., 2017).

#### 4.3.6 Dealing with missing data

Studies that did not report all data necessary to calculate an effect size were handled in one of three ways. First, authors were contacted in order to seek supplemental information to allow for standard calculations. For a subset of studies whose authors could not provide additional data, the *N* and mean but not *SD* figures were provided. However, *SD*s can be imputed for effect size calculations with continuous outcomes (Furukawa, Barbui, Cipriani, Brambilla, & Watanabe, 2006, Philbrook, Barrowman, & Garg, 2007, Stevens, 2011). For studies missing *SD* data, *SDs* were imputed (singly for YRE and traditional‐calendar students, by subject) based on other studies in the analytic sample with the same outcome (e.g., TerraNova or national percentile rank).^6^ Table 2 shows the studies in the third group: studies for which data for extracting a comparable effect size was not included in the study, was not available from the author, and could not be imputed.

**Table 2:** Characteristics of studies meeting criteria, but reporting noncomparable outcome data

Study author and year	Analysis and finding summary	State	Calendar structure	Weeks of summer	Grade level
*Analysis method that does not allow extraction of Hedges’ g or logged odds for synthesis*					
Anderson (2009)	Two‐level regression with interaction term for YRE/grade. Positive for both subjects; author estimated *d* 0.03–0.10	HI	Not standard	6	3, 4
Graves (2011)	Change in % at 25th, 50th, 75th percentiles from YRE and number of years on calendar. More negative than positive coefficients, sensitive to specification, about half significant	CA	Mixed	Mixed	All
Johnson (2005)	*T* test of difference in mean of % proficient (with no way to extract odds ratio); statistically insignificant findings in math and communication arts	MO		6	3, 4, 7, 8, 10, 11
Marlett (2007)	4‐way ANOVA with positive, insignificant effect for reading (no math analysis)	IL	45‐15		3, 8
Tittermary et al. (2013)	School‐level gain relative to predicted. Black students made faster gains, esp. in math, at YRE schools; as did Hispanic and economically disadvantaged students	VA	Mixed	“~6”	3–5, 7–8, 11
*Mixed subjects into single outcome variable*					
Beringer (2002)		Mixed	45‐15 and 45‐10	6	11
Corbett (2003)		AL	Mixed	Mixed	4
Stenvall and Stenvall (2001)		CA	Mixed	Mixed	Mixed
Wilmore and Slate (2012)		TX	Mixed	Mixed	5

*Note.* Data extracted from primary study documents.

Abbreviations: ANOVA,analysis of variance; YRE, year‐round education.

^a^We are indebted to the authors who shared additional, unpublished data for inclusion in this meta‐analysis.

#### 4.3.7 Assessment of heterogeneity

We tested for heterogeneity among the effect size estimates provided by the studies in our final sample using both *τ* ^2^ and *ω* ^2^. In RVE analysis using hierarchical weights, *ω* ^2^ is a measure of variation in within‐study (within‐cluster) estimates of effect. *τ* ^2^, instead, estimates variance between clusters, and is therefore more similar to the meta‐analytic measures of heterogeneity with which readers may be more familiar.

#### 4.3.8 Data synthesis

Hedges et al. (2010a) discuss the hierarchical dependence form of RVE as applying to multiple studies produced by the same lab. Our final sample has hierarchical dependence from multiple estimates (of different but not independent samples) from the same study, so the same type of correlation needs to be accounted for. We therefore use hierarchical weights in the RVE rather than the correlated effects weights (which are intended for addressing the dependence among multiple measures of the same outcome or group). Hedges et al. (2010a) find that 50 estimates from 10 studies leads to almost nominal results (0.944 to 0.957 for the nominal 95% confidence interval), with nearly nominal results for less‐balanced distributions of estimates, confirming that the YRE sample is large enough to produce valid RVE estimates. Additionally, our models made use of a small sample correction to both residuals and degrees of freedom in order to reduce the Type I error rate (Tipton, 2015). The RVE calculation of the meta‐regression coefficient only (i.e., the effect size value of interest) can be used with as few as 10 studies (Tanner‐Smith & Tipton, 2014). Our sample is therefore large enough to use RVE to estimate the effect size of YRE (but not to also estimate coefficients for any calendar or study characteristics as independent variables). Our final model, run separately for math and for reading, is an RVE meta‐regression calculation of the coefficient only, using the small sample correction and hierarchical weights. This calculation is conducted twice within each subject, once to produce a Hedges’ *g* estimate for continuous measures of achievement and once to produce an estimated odds ratio for dichotomous measures of proficiency.

#### 4.3.9 Subgroup analysis and investigation of heterogeneity

The analytic sample for this synthesis included 30 studies. Three sets of analyses were conducted on their effect sizes. First, we conducted a main effect calculation, using RVE to calculate a cross‐study weighted average (correctly accounting for correlated errors) for continuous and dichotomous outcomes in reading and math. We then conducted analyses of this same structure restricted only to estimates for low‐income students and only to estimates for minority students, because the literature on summer learning loss might predict YRE to provide greater benefit to historically disadvantaged students. We also conducted analysis of this structure divided by grade span, to assess whether there appear to be differential effects in elementary and middle schools.

Any difference in the effect of YRE for elementary‐aged children relative to middle school and high school may relate to differences in cognitive and memory development between elementary and middle school. Although much cognitive development occurs before school enrollment, memory function continues to develop during later childhood (Ghetti & Angelini, 2008; Lee, Wendelken, Bunge, & Ghetti, 2016; Ofen, 2012; Ofen et al., 2007; Rajan & Bell 2015). Notable among these changes is a shift from autobiographical to episodic memory (Pathman, Samson, Dugas, Cabeza, & Bauer, 2011). Not only does memory formation shift in this large sense during middle childhood, but in fact different facets of episodic memory develop at different rates (Picard, Cousin, Guillery‐Girard, Eustache, & Piolino, 2012; Shing & Lindenberger, 2011), as well as metacognitive changes, such as altered strategies for memory (Shing et al., 2010). These differences have implications for how children learn at different ages (Fandakova & Bunge, 2016; Ofen, Yu, & Chen, 2016; Prabhakar, Coughlin, & Ghetti, 2016; Shing and Brod, 2016). Importantly for this context, these differences in cognition and memory may mean that, simply put, even the shortened summer break typical in YRE calendars may still be too long to eliminate summer learning loss for students in grades K‐5. For example, a 6‐week summer may be too long for a 6‐year‐old student to show substantially increased recollection at the end of her summer break, and only a yet‐shorter summer would produce decreased summer learning loss for the youngest students. Because this is an unstudied question, we assess whether there are differences in YRE’s effect by grade span.

We deliberately conducted univariate subgroup analyses instead of meta‐regression with any independent variables because of the *N* of studies included for each measure. The number of estimates in this synthesis (never more than 20 studies for any math/reading continuous/dichotomous pairing) is not large enough to meet the guidelines for having an independent variable (in addition to the main effect estimate) in the RVE meta‐regression (see Tanner‐Smith & Tipton 2014). The same limitation precluded simultaneous consideration of multiple moderators, including looking across one issue of study design and another of student characteristic (e.g., matching type and calendar structure). Any 2 × 2 table of any paired set of moderators (e.g., of racial minority status and level of schooling) would average just five studies per cell. Because of this, we have retained subgroup analyses as our tool for descriptively comparing effect size estimates from studies and for students with different characteristics.

## 5 RESULTS

### 5.1 Description of studies

#### 5.1.1 Results of the search

Figure 1 illustrates the flow of documents during the search process. Initial searching identified 346 results, with another 153 found through footnote‐chasing, cited reference searching, and expert identification. Applying the four exclusion criteria to these results (reading abstract‐only) reduced the sample to 81 studies that were reviewed in full text to apply the same exclusion criteria and limit examination to studies of single‐track YRE. The quantitative meta‐analyses presented below are of a limited subset of this initial sample.^7^

In order to ensure coding quality, a second researcher coded 25% of search results with inter‐rater reliability of 90% and all nonmatched coding discussed until consensus was reached. A 25% sample of the full‐text reviews were also conducted by two researchers, with all differences resolved with full agreement on the final sample. The first author extracted the data for calculating effect sizes (both continuous and dichotomous outcomes) on two separate occasions (generally separated by several months) and calculated the effect size estimate and variance using each set of figures, achieving intrarater reliability over 0.96 and correcting all nonmatching estimates. After applying all restrictions, the resulting sample included 30 studies.

#### 5.1.2 Included studies

Table 1 shows the characteristics of the 30 studies included in our meta‐analytic calculations. It reveals variety in state, grades served, calendar structure, and summer length. Table 2 shows the characteristics of the nine studies that otherwise met inclusion criteria but had academic outcome data from which a comparable effect size estimate could not be extracted. Atypically, the majority of the studies in Tables 1 and 2 are dissertations. Published works, perhaps in order to increase their sample size to make statistically significant findings easier to achieve, tended to look at mixed single‐ and multitrack YRE. As a result, excluding mixed studies resulted in a final sample with three reports, two conference presentations, five articles, and 20 dissertations. We encourage readers interested in greater detail about the final sample, including achievement measures, identification strategy, and modeling to refer to Table 7.

Both tables illustrate the weak reporting of calendar structure and summer length in primary studies of YRE. Descriptively, it is of interest that Table 1 shows that two of the six negative Hedges’ *g* effect size estimates are from the only two studies of schools that retained an 8‐week break for summer, rather than a shorter break (with two more from schools with 6‐week breaks, and none for studies reporting schools with summer shortened to 5 or 4 weeks). The 30 studies examined predominantly 45‐10 or 45‐15 calendars serving students in grades 3–5. Only three studies included grades earlier than three, and only three studies examined high school students.

#### 5.1.3 Excluded studies

The descriptive features of the studies whose results could not be included in our meta‐analytic calculations are similar to those of the included studies. These nine studies are primarily of late elementary grades, conducted in a variety of states and with weak reporting of calendar structure and summer vacation length. Table 2 reveals that all of the statistically significant findings from excluded studies were of positive effects for single‐track YRE.

### 5.2 Risk of bias in included studies

For both dichotomous and continuous outcomes, Table 4 reveals important differences in estimates for analyses using differing identification strategies. Studies comparing YRE students to others in the same school district, county, or other geographic proximity show *g* estimates that are more than twice as large as those in the full sample of studies, although proficiency estimates are marginally smaller. Cohort comparison analyses produce larger‐magnitude effect size estimates in reading, but an insignificantly negative *g* estimate (near zero) in math. The results for studies using matching look very like the estimates across all identification strategies: insignificant estimates for dichotomous outcomes (though with slightly larger point estimates than for the full sample of studies), +0.09 for math, and +0.11 for reading. These patterns indicate that identification strategies do differentially introduce bias into the estimates of YRE’s effect.

**Table 3:** Average estimates of math and reading effect sizes for overall sample and subsamples, RVE

Sample		Hedges’ *g*, math	Odds ratio, math	Hedges’ *g*, reading	Odds ratio, reading
Full sample	Estimates	0.08*	1.03^c^	0.17**	0.96^c^
	95% CI	0.01, 0.15	0.68, 1.55	0.08, 0.26	0.73, 1.27
	*τ* ^2^	0.000	0.1557	0.0055	0.0775
	*ω* ^2^	0.0508	0.000	0.0217	0.000
Historically disadvantaged students					
Low‐SES	Estimates	0.06		0.13	
	95% CI	−0.04, 0.15		−0.07, 0.33	
	*τ* ^2^	0.0110		0.0227	
	*ω* ^2^	0.0127		0.000	
Minority	Estimates	0.13		0.10	
	95% CI	−0.05, 0.30		−0.04, 0.24	
	*τ* ^2^	0.0177		0.0056	
	*ω* ^2^	0.0196		0.0348	
Level of school					
Elementary^b^	Estimates	0.06	1.03	0.18*	0.89
	95% CI	−0.06, 0.17	0.64, 1.65	0.03, 0.32	0.68, 1.15
	*τ* ^2^	0.000	0.2218	0.0139	0.0503
	*ω* ^2^	0.1139	0.000	0.0350	0.0335
Middle^b^	Estimates	0.16*		0.14*	
	95% CI	0.05, 0.28		0.04, 0.25	
	*τ* ^2^	0.000		0.000	
	*ω* ^2^	0.0290		0.0023	

*Note:* Based on the number of estimates included, especially in the subsample analyses, random effects are probably inappropriate, despite the statistically significant heterogeneity present in the fixed‐effects models.

^a^Statistically significant heterogeneity among the estimates included in this model.

^b^ Elementary grades defined as K‐5, middle grades as 6–8.

^c^ Although Carl (2009) has the largest *N* of students, excluding Carl (2009) from analyses does not produce different conclusions about dichotomous outcomes at year‐round schools. The point estimates are shifted to 1.03 in reading and 1.22 in math, but remain insignificant.

+*p* < .10.

* *p* < .05.

** *p* < .01.

***p < .001.

**Table 4:** Sensitivity of estimates to identification strategy of primary studies

Sample		Hedges’ *g*, math	Odds ratio, math	Hedges’ *g*, reading	Odds ratio, reading
*Full sample*	Estimates	0.08*	1.03	0.17**	0.96
	95% CI	0.01, 0.15	0.68, 1.55	0.08, 0.26	0.73, 1.27
	*τ* ^2^	0.000	0.1557	0.0055	0.0775
	*ω* ^2^	0.0508	0.000	0.0217	0.000
*Identification strategy*					
Proximity(e.g., samecounty, district)	Estimates	0.20	0.85	0.36* ^,^ b	0.85
	95% CI	−0.14, 0.53	0.64, 1.13	0.09, 0.63	0.43, 1.68
	*τ* ^2^	0.000	0.1914	0.0159	0.0564
	*ω* ^2^	0.3997	0.000	0.0096	0.0073
Cohortcomparison	Estimates	−0.01	1.17	0.21	1.45+
	95% CI	−0.24, 0.23	0.58, 2.37	−0.13, 0.55	0.86, 2.42
	*τ* ^2^	0.000	0.0506	0.0063	0.0035
	*ω* ^2^	0.0442	0.000	0.0443	0.0184
Matching	Estimates	0.09	1.25	0.11* ^,^ b	1.17
	95% CI	−0.04, 0.21	0.22, 7.28	0.06, 0.17	0.39, 3.44
	*τ* ^2^	0.0069	0.000	0.0003	0.1132
	*ω* ^2^	0.0116	0.000	0.0082	0.0000

*Note:* Based on the number of estimates included, especially in the subsample analyses, random effects are probably inappropriate, despite the statistically significant heterogeneity present in the fixed‐effects models.

^a^Statistically significant heterogeneity among the estimates included in this model.

Abbreviations: CI, confidence interval; RVE, robust variance estimate.

^b^ Because of limited DF in RVE calculations, the *p* value may be untrustworthy.

^+^ *p* < .10.

* *p* < .05.

** *p* < .01.

***p < .001.

### 5.3 Synthesis of results

#### 5.3.1 Full‐sample effects

For each study that included multiple estimates we used inverse‐variance weights to calculate a single effect size for each study to display in Table 1. However, we used RVE meta‐regression (intercept only) with the small sample correction to combine all effect sizes across studies into an estimated effect size for single‐track YRE. Table 3 reveals that the RVE estimates of the effect of single‐track YRE differ for continuous and dichotomous outcomes. Effect sizes for mean performance are always positive and sometimes statistically significant. The odds ratios, on the other hand, are close to 1.0 indicating no average effect. This combination of overall effects estimates may indicate that the effect of YRE is in improving the performance (or diminishing the summer slide) of students below proficiency, but that possibility could not be explicitly tested with these data. The overall Hedges’ *g* estimates are large relative to the estimated size of summer learning loss (estimated at 0.11 in reading and 0.16 in math), but counter to expectations, the estimate for reading (0.17; 95% CI, 0.08–0.26) is larger than the estimate for math (0.08; 95% CI, 0.01–0.15) for the full sample. Both estimates reveal minimal underlying heterogeneity, with *τ* ^2^ values of 0.0055 in reading and 0 in math.

##### Heterogeneity

Recall that *τ* ^2^ estimates variance between clusters and is therefore similar to the meta‐analytic measures of heterogeneity with which readers may be more familiar. The estimates for *τ* ^2^ in RVE models of dichotomous outcomes are much larger than for Hedges’ *g.* This is not surprising, given how sensitive proficiency rates are to shifts in cut scores. For the mean difference analyses, estimates for *τ* ^2^ are in general quite small: zero for four of the estimates in Table 3, and never above 0.0227 (for math for low‐SES students), a pattern which is also evident in Tables 5, 6. The estimates can be transformed into *SD* estimates—estimates of how stable or varied the true effect is—for each model (Borenstein et al., 2009). Smaller estimates for *τ* ^2^ imply relatively narrow bands for the range of effect size estimates; for example, 95% of reading estimates would be expected to be between 0.02 and 0.32. Across specifications, nearly half of RVE analyses produce *τ* ^2^ values of zero, indicating a precise estimate with minimal variation in the underlying studies’ estimates.

**Table 5:** Preliminary analysis of effect of YRE based on calendar characteristics

Characteristic		Hedges’ *g*, math	Odds ratio, math	Hedges’ *g*, reading	Odds ratio, reading
*Calendar structure, RVE*					
45‐15	Estimates	0.23	0.58	0.32+	0.63
	95% CI	−0.18, 0.63	0.04, 8.87	−0.04, 0.67	0.04, 9.52
	*τ* ^2^	0.0065	1.1537	0.0211	0.000
	*ω* ^2^	0.0996	0.000	0.0513	0.2978
45‐10	Estimates	0.08	1.52	0.09*	1.13
	95% CI	−0.09, 0.25	0.94, 2.46	0.01, 0.18	0.58, 2.20
	*τ* ^2^	0.000	0.000	0.0000	0.000
	*ω* ^2^	0.000	0.0819	0.0070	0.0588
*Weeks of summer, weighted avg.*					
4		0.63	0.80	0.32	0.84
5		0.38	1.08	0.15	1.09
“4 to 6”		0.21		0.13	
6		0.16	1.17	0.08	1.02
7		0.16		0.14	
8		0.00		0.11	

Abbreviations: RVE, robust variance estimate; YRE, year‐round education.

**Table 6:** Growth outcome analyses

Study author	Growth measure	Math difference	Reading difference
Anderson	Student‐level growth in scale score, grade 3 to 4	+13.8	+6.91
Anderson	Student‐level growth in scale score, grade 4 to 5	+6.65	+4.44
Carl	Average of student‐level growth in scale score for nonmobile students 2005–2007, starting grades 3–6	+21.33	+10.86
McMillan	Student‐level 3‐year National Curve Equivalent gain scores, grades 3–5	+1.8	+0.01
Mitchell‐Hoefer	Cohort change in share proficient, tracking students who stayed in the same school	−1.0	−10
Ramos	Student‐level national percentile rank, fifth grade minus third grade	+5.165	+1.645
Thigpen	Grade 3 to 5 change in share of students proficient; student‐level analysis of students enrolled only in YRE or TR schools	+13.86	+5.68
Tittermary	Average SOL score compared with regression‐predicted score. Reported as within 10 points or lower/higher than predicted. Number is the share of students lower than predicted subtracted from the share higher than predicted		
	Black	+19% (45‐26)	+16% (29‐13)
	Latino/a	7% (33–40)	+7% (27‐20)
	FRPL	+13% (43‐29)	+6% (19‐13)
Tittermary	Share of YRE schools at which student SOL scores grew faster than the average of traditional schools		
	Overall	55%	42%
	Black students	65%	74%
	Latina/o students	53%	76%
	FRPL	42%	61%

*Note.* Data extracted from primary study documents.

Abbreviations: FRPL, free or reduced price lunch; SOL, Standards of Learning.

##### Effect by student characteristics

Given that summer learning loss is most evident among students from disadvantaged groups, the estimated effects for low‐income and minority students are unexpectedly about the same magnitude or smaller than for the full sample, and are not statistically significant. For low‐income students, we find an effect size of 0.06 in math (95% CI, −0.04 to 0.15) and 0.13 in reading (95% CI, −0.07 to 0.33). For minority students we find an effect size of 0.13 in math (95% CI, −0.05 to 0.30) and 0.10 in reading (95% CI, −0.04 to 0.24). That the estimated effect size is larger in math than in reading for the minority subsamples, and that it is larger than the full‐sample estimate, is more aligned with predictions. However, we hesitate to interpret too much based on this one of the four coefficients for historically disadvantaged students. In reading, the estimated effects in elementary grades (0.18; 95% CI, 0.03–0.32) and middle grades (0.14; 95% CI, 0.04–0.25) are very similar, with the *τ* ^2^ statistic indicating greater heterogeneity within the elementary estimates. In math, the apparent effect of single‐track YRE is greater in middle school than in the elementary grades (0.16; 95% CI, 0.05–0.28) versus 0.06 (95% CI, −0.06–0.17), both with *τ* ^2^ values of 0). This could be because elementary math skills like addition or multiplication may be more likely to be used during summer months than middle‐school math like algebra. In addition to estimates mostly smaller than for the full sample, the low‐SES and minority estimates show slightly elevated *τ* ^2^ values, indicating the least precision and greatest heterogeneity of any set of effect sizes. Given that estimates for subgroups are based on a small sample, they should not be interpreted as conclusive; but they do suggest that the conceptualization of YRE as particularly effective for historically disadvantaged students may be an over‐simplification of a more nuanced situation.

This set of findings aligns with recent research adding nuance to our understanding of summer learning loss (which YRE is primarily intended to decrease). Individual works are neither systematic nor conclusive, but have shown larger losses in elementary than middle grades (Atteberry & McEachin, 2019) and revealed that the magnitude of measured summer learning loss is sensitive to what test is used (von Hippel, Workman, & Downey, 2018). Particularly pertinent to our findings, recent work calls into question whether modern data actually shows low‐income students to exhibit summer learning loss at any greater magnitude than higher‐income students (von Hippel and Hamrock, 2019; Von Hippel, 2019), and introduces the possibility that students whose achievement grows the most during the year recede the most during summer (Koury, Justice, Jiang, & Logan, 2019; Kuhfeld, 2019). The effectiveness of single‐track YRE for historically disadvantaged students may warrant particular focus in future research.

##### Calendar characteristics

Despite the incomplete reporting of calendar structure and summer length, we conducted preliminary analyses of how calendar characteristics relate to study estimates. Table 5 reveals mostly insignificant estimates for dichotomous outcomes, which suggest that shorter summers and 2‐week rather than 3‐week breaks during semesters are more beneficial to students. The odds ratio estimates by calendar structure, from subsample RVE calculations, have large and overlapping confidence intervals, but the estimates for 45‐10 calendars are positive and for 45‐15 are negative. For continuous outcomes, the math estimate is, descriptively, almost three times as large from studies of 45‐15 calendars relative to 45‐10 calendars; and the reading estimate, though less precisely estimated, is of similar relative magnitude. For summer length, the small number of estimates from each number of weeks made separate RVE analyses inappropriate. Instead, Table 5 reveals inverse‐variance weighted means by length of summer vacation. For both subjects, the largest *g* estimate is for the shortest summer. In math, each increase in summer length is (descriptively) associated with a lower estimated effect size, seeming to indicate that as summer is shorter, summer learning loss does indeed diminish**.** This finding, moreover, seems to align with the implicit theory of YRE advocates mentioned above, that the length of the longest break from school may determine the extent of learning loss, with forgetting having a nonlinear relationship to break length. If further analyses reinforce this understanding, that would emphasize the importance of reduced length of longest break as the critical mechanism for mitigating learning loss.

###### Growth

Year‐over‐year growth is in several respects a better measure of policy effectiveness than achievement or proficiency. However, just seven of the studies in the final sample report a form of growth, so assessment of the relationship between YRE and growth must be considered tentative. Additionally, the studies have different growth‐related outcome variables—including school‐level change in percent proficient, cohort change in percent proficient, student‐level change in proficiency status, school‐level growth in mean score, student‐level growth in score, growth relative to predicted value—which makes producing an estimated average effect seem unwise. Instead, the individual study findings are summarized in Table 6. Across the outcome variable examined, the studies tend to find positive effects for single‐track year‐round calendars on student growth.

## 6 DISCUSSION

### 6.1 Summary of main results

#### 6.1.1 Consistent positive results for average achievement but not proficiency

Across analyses, single‐track YRE consistently shows no effect on dichotomous outcomes but shows a positive effect on average achievement in both reading and math. The estimates are relatively stable for elementary schools, middle schools, minority students, and low‐SES students, but vary slightly depending on calendar characteristics and studies’ identification strategies. These estimates for subgroups may be less precise because of the smaller number of studies included in each calculation, and other explanatory factors may not have been distributed randomly, as none of the primary studies employed an experimental design. Overall, though, the magnitude of achievement increase from single‐track YRE is comparable to the estimated magnitude of summer learning loss.

### 6.2 Overall completeness and applicability of evidence

There are two important analyses that could not be completed in as rigorous a method as would be preferred with the data available because of extensive under‐reporting of calendar characteristics. The summer vacation of schools in the final sample for this meta‐analysis ranges from as short as 4 weeks to a high of 8 weeks, with vacations as long as 10 weeks appearing in other studies that were excluded in this analysis. Given that a premise of YRE is that the shortened summer break combats summer learning loss, a strong theoretical case can be made that shortening summer break to only 20 weekdays would be expected to have a different impact on students than a summer break shortened but still 40–50 weekdays long. However, less than half of the studies in the final sample reported the length of the summer vacation (and did not combine schools with multiple summer lengths to produce a single estimate of effect), which precluded formal analysis of whether a shorter summer is more beneficial than a longer summer within single‐track year‐round calendars.

Similarly, only half of the studies indicated which calendar structure the year‐round schools being studied used (and did not combine schools with multiple calendar structures to produce a single estimate of effect). Again, a strong theoretical case can be made that the different calendar structures (30‐5, 45‐10, 45‐15, 60‐20, and 90‐30) would be expected to have a different impact on students and teachers. Perhaps students on a 60‐20 calendar need a few days of review after each 4‐week break, and so some instructional days are lost to review on that calendar structure. Perhaps, instead, students on a 30‐5 calendar have reduced attention because they get no lengthy breaks during the year and have a shorter summer than students on a traditional calendar. A 45‐10 calendar might combine the strengths or combine the weaknesses of the calendars with more‐ and less‐frequent breaks. Unfortunately, because so few studies clearly reported data on calendar structure and because those that did report structure almost exclusively followed two of the structures, we could conduct only a preliminary assessment of how calendar structure links with student achievement within year‐round schools.

### 6.3 Quality of the evidence

The studies included in this meta‐analysis reflect diversity in geography, grade, and calendar characteristics. However, relatively few studies used advanced analyses or quasiexperimental design. Tittermary et al. (2013) calculated school‐level gains relative to predicted achievement, and Graves (2009, 2010) used school fixed effects and school‐specific time trends. The other studies split among proximate comparison schools (in the treatment school’s city, district, or region), cohort designs comparing an untreated cohort to a treated cohort when schools changed calendars, and matched designs of various complexity, mostly matched at the school level. Recall that in our assessment of risk of bias in included studies, we found that studies using proximate comparisons produced larger estimates than the full sample, cohort comparisons a larger estimate in reading and a smaller in math, and matching protocols produced estimates very similar to the overall findings, at +0.09 for math and +0.11 for reading. The more methodologically advanced analyses do not necessarily produce estimates with greater validity for our specific research questions, but it is worthwhile to note the relationship between effect size and study design. The variation used to produce the estimates in Graves (2009, 2010) is based on schools’ changes in calendar during the period examined, and in fact just over two‐thirds of the schools that switched to single‐track YRE were switching from multitrack calendars, not from traditional calendars. Higher‐validity estimates could be achieved by future researchers making use of more rigorous methods and/or student‐level matching. Note that the quality of moderator analyses is likely lower than the main findings due to the smaller sample of estimates from primary studies included and the resultant simpler analytic model used. For instance, the finding that single‐track YRE has a greater effect for middle school than elementary school should be considered suggestive. With stated caveats regarding the magnitude and point estimate of effect; the pattern of evidence and the average of findings both point toward modest positive effects for single‐track YRE, with apparently wide generalizability based on the diverse populations analyzed by the studies in the final sample.

### 6.4 Limitations and potential biases in the review process

#### 6.4.1 U.S.‐centered findings

A small number of foreign‐language studies were examined, with the assistance of researchers fluent in the language of those studies. However, all searches were conducted in English. As a result, it is possible that studies of year‐round calendars in non‐English‐speaking nations were not retrieved. As a result, this review should be treated primarily as having implications for United States policymaking, secondarily for other nations with similar policies and culture (Canada, UK, Australia), and less so for other nations, particularly those with starting school calendars that differ from the 180‐day calendar in the United States. Descriptively, one of just two math mean differences below −0.04 is from the only study of Guam, which perhaps emphasizes the potential importance of cultural and policy differences that link with geographic differences.

##### Reviewers

The process of meta‐analysis uses extensive reasoning, not just objective assessment that produces homogenous conclusions (Chan, Macdonald, Carnevale, Steele, & Shrier, 2018). In this light, the authors acknowledge that they *a priori* thought that YRE was likely to have a (small) positive effect, based on its theory of action and on the findings of prior meta‐analyses. We do not believe, though, that this in any way influenced our research synthesis process, studies that were included, estimates that were calculated, or any other facet of the work.

### 6.5 Agreements and disagreements with other studies or reviews

Our main estimates align in direction and are similar in magnitude to prior meta‐analyses, when they examined single‐track calendars separately from multitrack calendars. Single‐track YRE seems to offset a large share of summer learning loss in both math and reading. In an early meta‐analysis, Kneese (1996) did not look at single‐track alone but found an effect of +0.11 to 0.2 *SDs* for YRE. Later, Cooper et al. (2003) estimated that single‐track YRE had an effect size of +0.16 on a merged cross‐subject academic achievement outcome. Cooper et al. (1996), quantifying summer learning loss, estimated that achievement declines by 0.16 *SDs* in math and 0.11 in reading. Our main findings of +0.17 *SDs* in reading and +0.08 *SDs* in math have different point estimates, but align with the magnitude of prior findings.^8^

## 7 AUTHORS’ CONCLUSIONS

Across all specifications, the estimated effect size for both math and reading is generally positive. Its magnitude and statistical significance, though, are sensitive to the specifications used in estimating the effect size. The main model finds a modest significant effect for math and a modest significant effect for reading, which is consistent with prior findings.

The summer learning loss literature would have predicted a larger effect in math than in reading, which these data do not show. However, the estimates do indicate that single‐track YRE outperforms traditional calendar education by approximately the same amount as Cooper et al.’s (1996) estimate of summer learning loss. In both subjects, the estimate is, though modest, large enough to be policy‐relevant. Prior analysis has found effect sizes in the 0.1–0.2 range to be important in education policy (Bloom, Hill, Black & Lipsey, 2008; Hill, Bloom, Black, & Lipsey, 2008; Lipsey & Wilson, 1993)—for example, the estimated effect was 0.11 for year‐long Title 1 programs (Borman & D’Agostino, 1996).

### 7.1 Implications for practice and policy

The central conclusion from analyzing 2001–2016 data is that single‐track YRE has a modest but positive effect on student achievement. The magnitude of the effect size is sensitive to the subsample analyzed and the model used, but it is positive in all specifications.

#### 7.1.1 Costs, opposition to, and challenges of YRE

The cost of transportation, food service, maintenance, staff, and other services for 180 instructional days is ostensibly the same in single‐track YRE and in traditional‐calendar schools. In some locations, there would be a moderate up‐front cost of needing to install air conditioning, so that existing school buildings could be used during summer months. Overall, though, the reform imposes relatively few costs; primarily because it involves reallocating existing resources across the full year. In an analysis of YRE in Virginia conducted for the state legislature, for example, the primary factor increasing cost was instructional costs during intersession, which averaged 3% of operating costs (Tittermary et al., 2013).

Schools or districts considering switching to YRE are likely to face opposition. Long summer vacations may remain popular. However, it is distinctly possible that such opposition would be temporary. This review did not focus on satisfaction, but in the course of reviewing documents that were not part of our final sample, we noticed that several different studies that examined satisfaction with and/or opinions of YRE. They seemed to follow a pattern of teacher, student, and parent opposition to a proposed switch to YRE, diminished dissatisfaction during initial implementation of YRE, and a preference for YRE after an adjustment period. Since we did not systematically retrieve studies of satisfaction, this pattern must be considered a tentative observation; but it indicates a different structure than permanent opposition or dissatisfaction among students, parents, or teachers on a mature year‐round calendar. A systematic assessment of this pattern would be an important component of proposals in favor of YRE.

Switching to YRE also requires overcoming implementation hurdles. The absence of a single long break does have drawbacks, including that there is no period for large maintenance/reconstruction work to be done, teachers do not have an extended period to engage in professional development or curricular reform, and teachers and other staff do not have an off‐season in which to earn secondary income. Teachers in YRE schools who are not residents of the districts in which they teach may be on a different vacation schedule from their own children, which could reduce applicants for teaching positions and/or increase turnover. In addition to these management challenges, YRE does not typically add any instructional time, resources, or techniques. YRE is intended to counter summer learning loss; it is unlikely to make strides (in achievement or in closing gaps) beyond that. It is possible for schools to provide supplementary instruction during the frequent vacation weeks, thereby providing extended‐year school only for students who are struggling—some YRE advocates are strongly in favor of such “intersession” instruction—but doing so increases costs and challenges. YRE as purely reallocation of 180 instructional days does include face administrative barriers, and does not introduce instructional time or resources.

#### 7.1.2 Overall assessment

Given the relatively low cost of adopting single‐track YRE, this analysis supports increased adoption of single‐track YRE. YRE appears able to counter much of the measured drop from summer learning loss. Additionally, the estimated effect of YRE on student achievement we find in this meta‐analysis is similar to the estimated impact on student achievement that would be expected from increasing teacher quality by one *SD* (Hanushek & Rivkin, 2010).

### 7.2 Implications for research

Findings that single‐track YRE has a greater effect for middle school than for elementary school need to be considered very tentative based on the smaller sample of studies and weaker model used in the grade‐span analyses. However, they are new, as none of the final sample’s studies compared effects across grades, and Cooper et al. (1996) only looked at secondary and elementary education. The possibility of a greater effect for middle school may point to the need for greater cognitive/educational research examining how long‐term memory develops during elementary grades. Although this interpretation could inform future research, because the observed difference in effect by grade is a very tentative finding, this branch of research is likely less vital than further examination of calendar characteristics. Consistent positive estimates for YRE, but only provisional information on effects by grade, by student characteristics, or by calendar structure is suggestive that future research should begin to focus on which types of single‐track YRE are most effective for which types of students. As evidence of single‐track YRE’s effect grows, it becomes increasingly important to understand the characteristics that increase its effectiveness. Future research should therefore report results in a way that allows for variation in calendar structure and summer length to be studied in greater depth and detail. There may be important differences in how different summer lengths and how 30‐5, 45‐10, 45‐15, and 90‐30 calendars impact teachers and students. Omitted calendar characteristics limit researchers’ ability to examine these important questions and we therefore argue that future research on YRE should clearly identify the length of the summer break and calendar structure.

## ROLES AND RESPONSIBILITIES

Who is responsible for the below areas? Please list their names:

- Content: Dan Fitzpatrick
- Systematic review methods: Dan Fitzpatrick
- Statistical analysis: Dan Fitzpatrick
- Information retrieval: Dan Fitzpatrick & Jason Burns

## SOURCES OF SUPPORT

The work reported here was supported in part by funding from the Campbell Collaboration and in part by funding from Michigan State University and the Education Policy Center at MSU. The opinions expressed here are those of the authors and do not represent the views of any other parties.

We would also like to acknowledge assistance at various stages of this work from Spyros Konstantopoulos, Terri Pigott, Josh Polanin, Carlton Fong, Gary Ritter, and David Pickup.

## DECLARATIONS OF INTEREST

None

## PLANS FOR UPDATING THE REVIEW

I will remain responsible for updating this review every five years or will be available to help Campbell in identifying a new researcher to conduct the update.

## Supporting information

Supplementary information

## Footnotes

1 This issue is further complicated by divergent terminology, including historically controlled study (Higgins et al., 2013), historically controlled cohort study (Reeves, Wells, & Waddington, 2017), and single group study design with historical comparison (Paulus et al., 2014).

2 Aside from cross‐national and cross‐cultural differences, the typical number of instructional days varies by country.

3 Some studies reported data for White, African American, and Hispanic students, others reported White and non‐White students. I use the term *minority* to refer to non‐White students throughout this paper, even though Whites were <50% of students at some of the schools studied.

4 Because only four estimates had combined treatment and control samples of <100 and none were under 50, standard guidelines would indicate that the small‐sample correction was not needed (Hedges, 1981). However, calculating Hedges’ *g* is a more conservative approach that introduces minimal disadvantages.

5 Even for two studies that appear in published form as well, we refer to the dissertation as the primary source document, because the dissertations include the data needed to calculate effect sizes, while publication page limits can preclude that. As later descriptive statistics illustrate, the studies of only single‐track YRE included smaller Ns than most publication outlets prefer; we suspect that this is a contributing factor in the tendency for published works to mix single‐ and multitrack schools’ achievement. Several authors of dissertations in final samples subsequently worked as school administrators, creating less of a career incentive to seek publication than doctoral students who matriculate to university positions.

6 Specifically, we imputed *SDs* for four studies: mean percentile on Stanford Diagnostic Reading Test (SDRT) for D’Alois (2005), the same value for national percentile rank for Malicsi (2003) and NCE score for McLean (2002), and TerraNova scores for Varner (2003). Where retrievable, we imputed using national figures or publicly available test statistics, rather than imputing based on the other studies (e.g., Trent, 2007 and McMillan, 2005 for TerraNova) in our sample. We also generated *SD* figures from other provided figures, for example, standard error (Cary, 2006) and *F* test results (Abakwue, 2011). Given that the four studies for which *SDs* constitute 2.5% of the weight in both math and reading, our findings are not sensitive to other reasonable values for these *SDs*.

7 Studies excluded as having no control group include one Master’s research project that compared an urban YRE school to a convenience nonequivalent control group of the rural school at which Schmidt (2011) worked.

8 The magnitude is also similar for the studies in our own final sample that report science (+0.11) or social studies (+0.13) outcomes, all for middle school grades (Fitzpatrick, 2019).

## APPENDIX A

See Table 7

**Table A1:** Measurement, identification strategy, and analysis characteristics of studies in final sample

Study author and year	Achievement measure	Identification strategy	Analytic approach
Abakwue (2011)	TCAP (standards‐based)	Demographically similar schools, geographically proximate	2‐group MANOVA for scores of 30 randomly selected students (/subject/schl)
Beazley (2001)	% proficient on district‐generated criterion‐referenced test	Cohort comparison within school; 3 years before change vs. first 3 years YRE	Descriptive analysis of means and 3‐year trends; descriptive comparison to district
Carl (2009)	WI Knowledge and Concepts Examination (criterion‐referenced)	Compared YRE schools’ performance to the balance of Milwaukee Public Schools	Year‐over‐year percent proficient; average growth in score
Cary (2006)	SOL, criterion‐referenced	Title 1 schools matched on FRPL and race	MANOVA
Coopersmith (2011)	TAKS (norm‐referenced) raw score	School‐level pairing within TEA campus comparison group, matched on ethnicity, economic status, LEP, and mobility	Independent samples *t* test (mean difference)
Crow (2009), Crow and Johnson (2010)	TAKS (standards‐referenced)	TEA campus comparison group matched on % African American, % Hispanic, % white, % economically disadvantaged, % LEP, % mobile (<10% differences)	Independent samples *t* test (mean difference)
D’Alois (2005)	% passing VA SOL, PALS; mean percentile SDRT	Cohort comparison within school of 1 year before and 1 year after conversion; comparison with other schools in city	*t* test of average percent passing and mean achievement
Evans (2007)	% Passing ISTEP+, criterion‐references	School‐level matching on FRPL, minority, ELL	*t* test of average percent passing
Ferguson (2001)	% passing SOL	Cohort comparison of 1 year before and 1 year after conversion	Descriptive comparison of % passing
Fritts‐Scott (2005)	ACTAAP Primary Benchmark Exam (criterion‐referenced) scaled score	School‐level within‐state 3‐stage 2:1 matching of nine YRE schools based on school size, %FRPL, grade span; % minority, region, district size; random selection	One‐way ANOVA for students enrolled at the same school for 3 years
Graves (2009, 2010)	Average student national percentile rank	Uses school fixed effects and school‐specific time trends in order to estimate the effect of within‐school differences in calendar type	Regression (OLS) with extensive controls; OLS with school fixed effects; OLS with school‐specific time trends (primary specification)
Helton (2001)	% Proficient FCAT	Twenty three schools matched to in‐district comparison school with similar FRPL	ANOVA controlling for FRPL and LEP
Kellems (2006), Oppel (2007)	% passing ISTEP+	Cohort comparison, 2 years before and after conversion.	Descriptive analysis of pass rates before and after calendar conversion
Lindsay‐Brown (2010)	PACT (norm‐referenced) and MAP	District‐level matching on % not ready for first grade and FRPL; schools randomly selected.	ANCOVA
Malicsi (2009)	Stanford 9 (norm‐referenced)	Cohort comparison within schools, omitting the year in which the calendar was changed and 1 other; 2 years before on each side of policy change	Descriptive analysis of means of percentile rank stanine and comparison of those means to the district‐level means
Marks (2006)	TCAP (standards‐based)	Cohort comparison within school; 1 year before change vs. first 2 years YRE	Repeated measure ANOVA
McLean (2002)	TCAP (norm‐referenced) NCE.	Cohort comparison within school; also year‐over year NCE change within cohorts across YRE/traditional calendar years	Descriptive and trend analysis
McMillan (2005)	TCAP TerraNova (norm‐ and criterion‐referenced items)	School‐level matching on FRPL, rurality, and % minority	Independent samples *t* test (mean difference) of NCE (national curve equivalent) score
Merrill (2012)	ISAT (standards‐based) standard scores	Within‐district comparison schools matched based on % African American and % FRPL	2‐way between subjects factorial ANOVA
Mitchell‐Hoefer (2010)	% proficient, PACT	Within‐district comparison school based on Title I status	*Z* test of percent proficient, estimated separately for three consecutive years
Moore (2002), Moore and Verstegen (2004)	SOL (criterion‐referenced) and Stanford 9 (norm‐referenced)	School‐within‐a‐school with parent opt‐in. Comparable on descriptive characteristics, but treated group slightly less likely to receive FRPL and more likely to live with both parents	*t* test (mean difference)
Ramos (2006, 2011)	National Percentile Rank on ITBS, CAT‐5, and ISAT	Three school‐within‐schools	*t* test; ANCOVA controlling for gender, ethnicity, gifted, IEP, FRPL
Schumacher (2015)	% meeting standards, Nebraska State Assessments	Within‐district match based on % FRPL	One‐way ANOVA
Sexton (2003)	SOL (criterion‐referenced)	School‐within‐a‐school	One‐way ANCOVA controlling for 5th‐grade Degrees of Reading Power scores and attendance for non‐IEP students
Thigpen (2004)	% Proficient, Mississippi Curriculum Test	Within the same district; similar rates of FRPL, minority, and low‐performing students	*χ* ^2^ analysis of mean % proficient in 3 consecutive years, only for the students who remained on the same calendar all 3 years
Thomas (2002)	TLI scores from TAAS (criterion‐referenced)	Four treatment schools matched to TEA campus comparison group on ethnicity, %ED, % mobility, % LEP.	ANOVA controlling for ED, ethnicity, gender, and school size
Trent (2007)	TCAP TerraNova (norm‐ and criterion‐referenced items)	Counties selected based on similar rurality, % FRPL, rurality, ethnicity	Independent samples *t* test (mean difference) of NCE (national curve equivalent) score for students enrolled at the same school for 3 years
Varner (2003)	TerraNova median percentile rank	Within‐district comparison schools, comparable on % African American and % FRPL	Descriptive analysis of means and 4‐year trend
Wilmore and Slate (2012), Wilmore‐Dafonte (2013)	TAKS (standards‐referenced)	2:1 match from campus comparison group using % Black, % Hispanic, % White, %ED, % LEP, and % mobile	MANOVA and follow‐up ANOVA
Winkelmann (2010)	% Passing ISAT	Within‐Chicago match based on city region, enrollment total, and low‐income %	Paired *t* tests of mean % passing

Abbreviations: ACTAAP, Arkansas Comprehensive Testing Assessment, and Accountability Program; ANCOVA, analysis of covariance; ANOVA, analysis of variance; CAT‐5, California Achievement Test‐5; FRPL, free or reduced price lunch; ISAT, Idaho Standards Assessment Test; ISAT, Illinois Standards Achievement Test; ITBS, Iowa Test of Basic Skills; MANOVA, multivariate analysis of variance; MAP, Measures of Academic Progress; OLS, ordinary least squares; PACT, Palmetto Achievement Challenge Test; SDRT, Stanford Diagnostic Reading Test; SOL, Standards of Learning; TAAS, Texas Assessment of Academic Skills; TAKS, Texas Assessment of Knowledge and Skills;TCAP, Tennessee Comprehensive Assessment Program; TLI, Texas Learning Index.
