# Supplementary material for: Single‐track year‐round education for improving academic achievement in U.S. K‐12 schools: Results of a meta‐analysis
Source: Campbell Syst Rev. 2019 Sep 24;15(3):e1053. doi: 10.1002/cl2.1053 (PMC8356518; doi:10.1002/cl2.1053)
Supplement: Supplementary file 1 — Supplementary information [file CL2-15-e1053-s001.docx]

# Online supplements

List of online supplements

1. Database Search logs

**DATABASE SEARCH LOG FOR** **ERIC**

Date of Search: 7/17/17

Database supplier: ProQuest

**DATABASE-SPECIFIC SEARCH METHODS**

Search terms: ("year round education" OR "year round school" OR "year round school*") OR (("modified calendar" OR "alternative calendar" OR "balanced calendar" OR "year-round calendar") AND school*) OR ("alternative school calendar" OR "balanced school calendar" OR "modified school calendar")

Key words/index terms/etc. used: subject code of “Year Round Schools”

Fields searched: Anywhere

Database-specific restrictions or filters: Education Level field restricted to those covering grades K-12

Internal Code for Search:

("year round education" OR "year round school" OR "year round school*") OR (("modified calendar" OR "alternative calendar" OR "balanced calendar" OR "year-round calendar") AND school*) OR ("alternative school calendar" OR "balanced school calendar" OR "modified school calendar") OR su("Year Round Schools")

Comments on strategy:

No additional comments.

**RESULTS**

Retrieved records (including duplicates): 40

Studies’ full text retrieved (including duplicates): 14

**DATABASE SEARCH LOG FOR** **Education Full Text**

Date of Search: 7/17/17

Database supplier: EBSCOhost

**DATABASE-SPECIFIC SEARCH METHODS**

Search terms: “year round education” OR “year round school” OR “year round school*”) OR ((“modified calendar” OR “alternative calendar” OR “balanced calendar” OR “year-round calendar”) AND school*) OR (“alternative school calendar” OR “balanced school calendar” OR “modified school calendar”

Key words/index terms/etc. used: Subject Headings: year-round schools, summer vacations (Schools)

Fields searched: Subject terms where pertinent; otherwise, no selection

Database-specific restrictions or filters: Used Boolean/Phrase Search Mode; checked box for “apply equivalent subjects”; excluded source type “magazines”; excluded source type “book reviews”

Internal Code for Search:

"SU year-round schools OR SU summer vacations (Schools) OR ( “year round education” OR “year round school” OR “year round school*” ) OR ( (“modified calendar” OR “alternative calendar” OR “balanced calendar” OR “year-round calendar”) AND school* ) OR ( “alternative school calendar” OR “balanced school calendar” OR “modified school calendar” )"

Comments on strategy:

No additional comments.

**RESULTS**

Retrieved records (including duplicates): 43

Studies’ full text retrieved (including duplicates): 4

**DATABASE SEARCH LOG FOR** **Education Admin Abstracts**

Date of Search: 7/17/17

Database supplier: EBSCOhost

**DATABASE-SPECIFIC SEARCH METHODS**

Search terms: “year round education” OR “year round school” OR “year round school*”) OR ((“modified calendar” OR “alternative calendar” OR “balanced calendar” OR “year-round calendar”) AND school*) OR (“alternative school calendar” OR “balanced school calendar” OR “modified school calendar”)

Key words/index terms/etc. used: Subject Terms: year-round school, summer vacations (Schools)

Fields searched: Subject terms where pertinent; otherwise, all text fields

Database-specific restrictions or filters: Used Boolean/Phrase Search Mode; excluded source type “magazines”; checked box for “apply equivalent subjects”

Internal Code for Search:

"SU year-round schools OR SU summer vacations (Schools) OR ( “year round education” OR “year round school” OR “year round school*” ) OR ( (“modified calendar” OR “alternative calendar” OR “balanced calendar” OR “year-round calendar”) AND school* ) OR ( “alternative school calendar” OR “balanced school calendar” OR “modified school calendar” )"

Comments on strategy:

No additional comments.

**RESULTS**

Retrieved records (including duplicates): 11

Studies’ full text retrieved (including duplicates): 4

**DATABASE SEARCH LOG FOR** **PsycINFO with PsycArticles**

Date of Search: 7/19/17

Database supplier: ProQuest

**DATABASE-SPECIFIC SEARCH METHODS**

Search terms: "year round education" OR "year round school" OR "year round schools" OR "year round schooling" OR "year-round education" OR "year-round school" OR "year-round schools" OR "year-round schooling" (("modified calendar" OR "alternative calendar" OR "balanced calendar" OR "year-round calendar" OR "year round calendar") AND (school OR schools OR schooling)) "alternative school calendar" OR "balanced school calendar" OR "modified school calendar"

Key words/index terms/etc. used: subject: “Year Round Schools”

Fields searched: Anywhere, including full text

Database-specific restrictions or filters: Age group: childhood, school age, or adolescence

Internal Code for Search:

("year round education" OR "year round school" OR "year round schools" OR "year round schooling" OR "year-round education" OR "year-round school" OR "year-round schools" OR "year-round schooling" (("modified calendar" OR "alternative calendar" OR "balanced calendar" OR "year-round calendar" OR "year round calendar") AND (school OR schools OR schooling)) "alternative school calendar" OR "balanced school calendar" OR "modified school calendar") OR su("Year Round Schools")

Comments on strategy:

Asterisks and/or seeking both “year-round” and “year round” from a single query was not working, so I used the longer search string

**RESULTS**

Retrieved records (including duplicates): 13

Studies’ full text retrieved (including duplicates): 7

**DATABASE SEARCH LOG FOR** **ProQuest Dissertations & Theses Global**

Date of Search: 7/19/17

Database supplier: ProQuest

**DATABASE-SPECIFIC SEARCH METHODS**

Search terms: ("year round education" OR "year round school" OR "year round school*") OR (("modified calendar" OR "alternative calendar" OR "balanced calendar" OR "year-round calendar") AND school*) OR ("alternative school calendar" OR "balanced school calendar" OR "modified school calendar"))

Key words/index terms/etc. used:

1. Key word/index terms: "Academic achievement in year-round school" OR "Tradition vs. year-round school" OR "Academic progress in a year-round school"
2. Subject: "year round school"

Fields searched: All but full text

Database-specific restrictions or filters: None

Internal Code for Search:

su("year round school") OR diskw("Academic achievement in year-round school" OR "Tradition vs. year-round school" OR "Academic progress in a year-round school") OR all(("year round education" OR "year round school" OR "year round school*") OR (("modified calendar" OR "alternative calendar" OR "balanced calendar" OR "year-round calendar") AND school*) OR ("alternative school calendar" OR "balanced school calendar" OR "modified school calendar"))

Comments on strategy:

No additional comments.

**RESULTS**

Retrieved records (including duplicates): 70

Studies’ full text retrieved (including duplicates): 29

**DATABASE SEARCH LOG FOR** **Web of Science**

Date of Search: 7/19/17

Database supplier:

**DATABASE-SPECIFIC SEARCH METHODS**

Search terms: "year round education" OR "year round school" OR "year round school*" OR (("modified calendar" OR "alternative calendar" OR "balanced calendar" OR "year-round calendar") AND school*) OR "alternative school calendar" OR "balanced school calendar" OR "modified school calendar"

Key words/index terms/etc. used: None

Fields searched: Title and Subject

Database-specific restrictions or filters: None

Internal Code for Search:

[TS=("year round education" OR "year round school" OR "year round school*" OR (("modified calendar" OR "alternative calendar" OR "balanced calendar" OR "year-round calendar") AND school*) OR "alternative school calendar" OR "balanced school calendar" OR "modified school calendar")] OR [TI=("year round education" OR "year round school" OR "year round school*" OR (("modified calendar" OR "alternative calendar" OR "balanced calendar" OR "year-round calendar") AND school*) OR "alternative school calendar" OR "balanced school calendar" OR "modified school calendar")]

Comments on strategy:

No additional comments.

**RESULTS**

Retrieved records (including duplicates): 19

Studies’ full text retrieved (including duplicates): 7

**DATABASE SEARCH LOG FOR** **ProQuest Research Library**

Date of Search: 7/19/17

Database supplier: ProQuest

**DATABASE-SPECIFIC SEARCH METHODS**

Search terms: "year round education" OR "year round school" OR "year round school*" OR (("modified calendar" OR "alternative calendar" OR "balanced calendar" OR "year-round calendar") AND school*) OR "alternative school calendar" OR "balanced school calendar" OR "modified school calendar"

Key words/index terms/etc. used: subject of "year-round school"

Fields searched: All but full text

Database-specific restrictions or filters: Exclude source types: Newspapers, Trade Journals, Magazines

Internal Code for Search:

su("year-round school") OR all("year round education" OR "year round school" OR "year round school*" OR (("modified calendar" OR "alternative calendar" OR "balanced calendar" OR "year-round calendar") AND school*) OR "alternative school calendar" OR "balanced school calendar" OR "modified school calendar")

Comments on strategy:

No additional comments.

**RESULTS**

Retrieved records (including duplicates): 18

Studies’ full text retrieved (including duplicates): 1

**DATABASE SEARCH LOG FOR** **PsycExtra**

Date of Search: 7/19/17

Database supplier: ProQuest

**DATABASE-SPECIFIC SEARCH METHODS**

Search terms: "year round education" OR "year round school" OR "year round school*" OR (("modified calendar" OR "alternative calendar" OR "balanced calendar" OR "year-round calendar") AND school*) OR "alternative school calendar" OR "balanced school calendar" OR "modified school calendar"

Key words/index terms/etc. used: None

Fields searched: All but full text

Database-specific restrictions or filters: Age group: childhood, school age, or adolescence

Internal Code for Search:

all("year round education" OR "year round school" OR "year round school*" OR (("modified calendar" OR "alternative calendar" OR "balanced calendar" OR "year-round calendar") AND school*) OR "alternative school calendar" OR "balanced school calendar" OR "modified school calendar")

Comments on strategy:

No additional comments.

**RESULTS**

Retrieved records (including duplicates): 1

Studies’ full text retrieved (including duplicates): 0

**DATABASE SEARCH LOG FOR** **PolicyFile**

Date of Search: 7/19/17

Database supplier: ProQuest

**DATABASE-SPECIFIC SEARCH METHODS**

Search terms: "year round education" OR "year round school" OR "year round school*" OR (("modified calendar" OR "alternative calendar" OR "balanced calendar" OR "year-round calendar") AND school*) OR "alternative school calendar" OR "balanced school calendar" OR "modified school calendar"

Key words/index terms/etc. used: none

Fields searched: All fields

Database-specific restrictions or filters: none

Internal Code for Search:

all("year round education") OR all("year round school") OR all("year round school*") OR ((all("modified calendar") OR all("alternative calendar") OR all("balanced calendar") OR all("year-round calendar")) AND all(school*)) OR all("alternative school calendar") OR all("balanced school calendar") OR all("modified school calendar")

Comments on strategy:

No additional comments.

**RESULTS**

Retrieved records (including duplicates): 2

Studies’ full text retrieved (including duplicates): 0

**DATABASE SEARCH LOG FOR** **International Bibliography of the Social Sciences**

Date of Search: 7/19/17

Database supplier: ProQuest

**DATABASE-SPECIFIC SEARCH METHODS**

Search terms: "year round education" OR "year round school" OR "year round school*" OR (("modified calendar" OR "alternative calendar" OR "balanced calendar" OR "year-round calendar") AND school*) OR "alternative school calendar" OR "balanced school calendar" OR "modified school calendar"

Key words/index terms/etc. used: subject “year round schools”

Fields searched: e.g. Full Text, or Abstract, or All But Full Text

Database-specific restrictions or filters: None

Internal Code for Search:

su("Year round school") OR ("year round education" OR "year round school" OR "year round school*" OR (("modified calendar" OR "alternative calendar" OR "balanced calendar" OR "year-round calendar") AND school*) OR "alternative school calendar" OR "balanced school calendar" OR "modified school calendar")

Comments on strategy:

No additional comments.

**RESULTS**

Retrieved records (including duplicates): 25

Studies’ full text retrieved (including duplicates): 2

**DATABASE SEARCH LOG FOR** **Sociological Abstracts**

Date of Search: 7/19/17

Database supplier: ProQuest

**DATABASE-SPECIFIC SEARCH METHODS**

Search terms: "year round education" OR "year round school" OR "year round school*" OR (("modified calendar" OR "alternative calendar" OR "balanced calendar" OR "year-round calendar") AND school*) OR "alternative school calendar" OR "balanced school calendar" OR "modified school calendar"

Key words/index terms/etc. used: none

Fields searched: Anywhere

Database-specific restrictions or filters: none

Internal Code for Search:

"year round education" OR "year round school" OR "year round school*" OR (("modified calendar" OR "alternative calendar" OR "balanced calendar" OR "year-round calendar") AND school*) OR "alternative school calendar" OR "balanced school calendar" OR "modified school calendar"

Comments on strategy:

No additional comments.

**RESULTS**

Retrieved records (including duplicates): 15

Studies’ full text retrieved (including duplicates): 1

**DATABASE SEARCH LOG FOR** **EconLit**

Date of Search: 7/20/17

Database supplier: ProQuest

**DATABASE-SPECIFIC SEARCH METHODS**

Search terms: "year round education" OR "year round school" OR "year round school*" OR (("modified calendar" OR "alternative calendar" OR "balanced calendar" OR "year-round calendar") AND school*) OR "alternative school calendar" OR "balanced school calendar" OR "modified school calendar"

Key words/index terms/etc. used: none

Fields searched: Anywhere

Database-specific restrictions or filters: None

Internal Code for Search:

"year round education" OR "year round school" OR "year round school*" OR (("modified calendar" OR "alternative calendar" OR "balanced calendar" OR "year-round calendar") AND school*) OR "alternative school calendar" OR "balanced school calendar" OR "modified school calendar"

Comments on strategy:

No additional comments.

**RESULTS**

Retrieved records (including duplicates): 10

Studies’ full text retrieved (including duplicates): 6

**DATABASE SEARCH LOG FOR** **Social Services Abstracts**

Date of Search: 7/20/17

Database supplier: ProQuest

**DATABASE-SPECIFIC SEARCH METHODS**

Search terms: "year round education" OR "year round school" OR "year round school*" OR (("modified calendar" OR "alternative calendar" OR "balanced calendar" OR "year-round calendar") AND school*) OR "alternative school calendar" OR "balanced school calendar" OR "modified school calendar"

Key words/index terms/etc. used: none

Fields searched: Anywhere

Database-specific restrictions or filters: none

Internal Code for Search:

"year round education" OR "year round school" OR "year round school*" OR (("modified calendar" OR "alternative calendar" OR "balanced calendar" OR "year-round calendar") AND school*) OR "alternative school calendar" OR "balanced school calendar" OR "modified school calendar"

Comments on strategy:

No additional comments.

**RESULTS**

Retrieved records (including duplicates): 8

Studies’ full text retrieved (including duplicates): 0

**DATABASE SEARCH LOG FOR** **PRISMA**

Date of Search: 7/20/17

Database supplier: ProQuest

**DATABASE-SPECIFIC SEARCH METHODS**

Search terms: "year round education" OR "year round school" OR "year round school*" OR (("modified calendar" OR "alternative calendar" OR "balanced calendar" OR "year-round calendar") AND school*) OR "alternative school calendar" OR "balanced school calendar" OR "modified school calendar"

Key words/index terms/etc. used: none

Fields searched: Anywhere

Database-specific restrictions or filters: none

Internal Code for Search:

"year round education" OR "year round school" OR "year round school*" OR (("modified calendar" OR "alternative calendar" OR "balanced calendar" OR "year-round calendar") AND school*) OR "alternative school calendar" OR "balanced school calendar" OR "modified school calendar"

Comments on strategy:

No additional comments.

**RESULTS**

Retrieved records (including duplicates): 1

Studies’ full text retrieved (including duplicates): 0

**DATABASE SEARCH LOG FOR** **PAIS Index**

Date of Search: 7/20/17

Database supplier: ProQuest

**DATABASE-SPECIFIC SEARCH METHODS**

Search terms: "year round education" OR "year round school" OR "year round school*" OR (("modified calendar" OR "alternative calendar" OR "balanced calendar" OR "year-round calendar") AND school*) OR "alternative school calendar" OR "balanced school calendar" OR "modified school calendar"

Key words/index terms/etc. used: subject “school year”

Fields searched: Anywhere

Database-specific restrictions or filters: none

Internal Code for Search:

su.Exact("school year") OR ("year round education" OR "year round school" OR "year round school*" OR (("modified calendar" OR "alternative calendar" OR "balanced calendar" OR "year-round calendar") AND school*) OR "alternative school calendar" OR "balanced school calendar" OR "modified school calendar")

Comments on strategy:

No additional comments.

**RESULTS**

Retrieved records (including duplicates): 7

Studies’ full text retrieved (including duplicates): 0

**DATABASE SEARCH LOG FOR** **Google Scholar**

Date of Search: 7/20/17

Database supplier: Google

**DATABASE-SPECIFIC SEARCH METHODS**

Search terms:

1. "year round education" OR "year round school" OR "year round schools" OR "year round schooling" OR "year-round education" OR "year-round school" OR "year-round schools" OR "year-round schooling"
2. ("modified calendar" OR "alternative calendar" OR "balanced calendar" OR "year-round calendar" OR "year round calendar") AND (school OR schools OR schooling)
3. "alternative school calendar" OR "balanced school calendar" OR "modified school calendar"

Key words/index terms/etc. used: None

Fields searched: Title

Database-specific restrictions or filters: none

Internal Code for Search:

1. allintitle: "year round education" OR "year round school" OR "year round schools" OR "year round schooling" OR "year-round education" OR "year-round school" OR "year-round schools" OR "year-round schooling"
2. allintitle: ("modified calendar" OR "alternative calendar" OR "balanced calendar" OR "year-round calendar" OR "year round calendar") AND (school OR schools OR schooling)
3. allintitle: "alternative school calendar" OR "balanced school calendar" OR "modified school calendar"

Comments on strategy:

Character limits on queries meant that I had to do three separate searches in order to fit all results. Additionally, longer strings were required because Google did not apply asterisks as academic search tools usually do, and produced different results for searches with and without dashes in “year-round”.

**RESULTS**

Retrieved records (including duplicates): 202

Studies’ full text retrieved (including duplicates): 32

**DATABASE SEARCH LOG FOR** **Periodicals Index Online**

Date of Search: 7/20/17

Database supplier: ProQuest

**DATABASE-SPECIFIC SEARCH METHODS**

Search terms: ("year round education" OR "year round school" OR "year round schools" OR "year round schooling" OR "year-round education" OR "year-round school" OR "year-round schools" OR "year-round schooling" OR (("modified calendar" OR "alternative calendar" OR "balanced calendar" OR "year-round calendar" OR "year round calendar") AND (school OR schools OR schooling)) OR "alternative school calendar" OR "balanced school calendar" OR "modified school calendar")

Key words/index terms/etc. used: none

Fields searched: Anywhere

Database-specific restrictions or filters: none

Internal Code for Search:

(all("year round education") OR all("year round school") OR all("year round schools") OR all("year round schooling") OR all("year-round education") OR all("year-round school") OR all("year-round schools") OR all("year-round schooling") OR ((all("modified calendar") OR all("alternative calendar") OR all("balanced calendar") OR all("year-round calendar") OR all("year round calendar")) AND (all(school) OR all(schools) OR all(schooling))) OR all("alternative school calendar") OR all("balanced school calendar") OR all("modified school calendar"))

Comments on strategy:

Longer search string employed to confirm that there are still no results post-2000.

**RESULTS**

Retrieved records (including duplicates): 0

Studies’ full text retrieved (including duplicates): 0

**DATABASE SEARCH LOG FOR** **Sociology Database**

Date of Search: 7/31/17

Database supplier: Proquest

**DATABASE-SPECIFIC SEARCH METHODS**

Search terms: "year round education" OR "year round school" OR "year round school*" OR (("modified calendar" OR "alternative calendar" OR "balanced calendar" OR "year-round calendar") AND school*) OR "alternative school calendar" OR "balanced school calendar" OR "modified school calendar"

Key words/index terms/etc. used: None

Fields searched: Anywhere

Database-specific restrictions or filters: None

Internal Code for Search:

"year round education" OR "year round school" OR "year round school*" OR (("modified calendar" OR "alternative calendar" OR "balanced calendar" OR "year-round calendar") AND school*) OR "alternative school calendar" OR "balanced school calendar" OR "modified school calendar"

Comments on strategy:

No additional comments.

**RESULTS**

Retrieved records (including duplicates): 30

Studies’ full text retrieved (including duplicates): 0

**DATABASE SEARCH LOG FOR** **Google (not Scholar)**

Date of Search: 8/1/17

Database supplier:

**DATABASE-SPECIFIC SEARCH METHODS**

"year round education" OR "year round school" OR (("modified calendar" OR "alternative calendar" OR "balanced calendar" OR "year-round calendar") AND (school OR schools OR schooling)) OR "alternative school calendar" OR "balanced school calendar" OR "modified school calendar" OR "year-round schools"

Key words/index terms/etc. used: N/A

Fields searched: all

Database-specific restrictions or filters: None

Internal Code for Search:

Comments on strategy:

As with Scholar, wild card characters did not work properly, so I adjusted the standard search string, while operating within Googles 32-word limit in query text.

**RESULTS**

Search Results (including duplicates): 294

Retrieved records (i.e. links opened): 294

Studies’ full text retrieved (including duplicates): 11
